# Extracellular Vesicles Derived from Acidified Metastatic Melanoma Cells Stimulate Growth, Migration, and Stemness of Normal Keratinocytes

**DOI:** 10.3390/biomedicines10030660

**Published:** 2022-03-12

**Authors:** Maxim L. Bychkov, Artem V. Kirichenko, Irina N. Mikhaylova, Alexander S. Paramonov, Evgeny V. Yastremsky, Mikhail P. Kirpichnikov, Mikhail A. Shulepko, Ekaterina N. Lyukmanova

**Affiliations:** 1 Shemyakin-Ovchinnikov Institute of Bioorganic Chemistry, Russian Academy of Sciences, 119997 Moscow, Russia; maksim.bychkov@gmail.com (M.L.B.); bittert@mail.ru (A.V.K.); a.s.paramonov@gmail.com (A.S.P.); kirpichnikov@inbox.ru (M.P.K.); mikhailshulepko@gmail.com (M.A.S.); 2 Moscow Institute of Physics and Technology, State University, 141701 Dolgoprudny, Russia; 3 Federal State Budgetary Institution “N.N. Blokhin National Medical Research Center of Oncology”, Ministry of Health of Russia, 115548 Moscow, Russia; irmikhaylova@gmail.com; 4 National Research Center “Kurchatov Institute”, Akademika Kurchatova pl. 1, 123182 Moscow, Russia; e.yastremsky@gmail.com; 5 Shubnikov Institute of Crystallography of Federal Scientific Research Centre “Crystallography and Photonics” of Russian Academy of Sciences, Leninskiy Prospect 59, 119333 Moscow, Russia; 6 Interdisciplinary Scientific and Educational School of Moscow University “Molecular Technologies of the Living Systems and Synthetic Biology”, Faculty of Biology, Lomonosov Moscow State University, 119234 Moscow, Russia

**Keywords:** melanoma, extracellular vesicles, miRNA, mRNA, SNAI, cancer, metastasis, migration, cytokines, adhesion factors

## Abstract

Metastatic melanoma is a highly malignant tumor. Melanoma cells release extracellular vesicles (EVs), which contribute to the growth, metastasis, and malignancy of neighboring cells by transfer of tumor-promoting miRNAs, mRNA, and proteins. Melanoma microenvironment acidification promotes tumor progression and determines EVs’ properties. We studied the influence of EVs derived from metastatic melanoma cells cultivated at acidic (6.5) and normal (7.4) pH on the morphology and homeostasis of normal keratinocytes. Acidification of metastatic melanoma environment made EVs more prooncogenic with increased expression of prooncogenic mi221 RNA, stemless factor CD133, and pro-migration factor SNAI1, as well as with downregulated antitumor mir7 RNA. Incubation with EVs stimulated growth and migration both of metastatic melanoma cells and keratinocytes and changed the morphology of keratinocytes to stem-like phenotype, which was confirmed by increased expression of the stemness factors KLF and CD133. Activation of the AKT/mTOR and ERK signaling pathways and increased expression of epidermal growth factor receptor EGFR and SNAI1 were detected in keratinocytes upon incubation with EVs. Moreover, EVs reduced the production of different cytokines (IL6, IL10, and IL12) and adhesion factors (sICAM-1, sICAM-3, sPecam-1, and sCD40L) usually secreted by keratinocytes to control melanoma progression. Bioinformatic analysis revealed the correlation between decreased expression of these secreted factors and worse survival prognosis for patients with metastatic melanoma. Altogether, our data mean that metastatic melanoma EVs are important players in the transformation of normal keratinocytes.

## 1. Introduction

Metastatic melanoma is a highly malignant tumor whose therapy is hampered by inflammatory, hypoxic, and acidic milieu promoting tumor growth and invasion [1,2]. Both melanoma growth and metastasis are regulated not only by intrinsic cellular mechanisms, such as the PI3K/AKT/mTOR pathway [3], but also by cross-talk between melanoma cells, neighboring cells, and extracellular matrix (ECM) by means of extracellular vesicles (EVs) [4]. EVs are membrane-coated cell-derived particles and can be classified as large vesicles (200–800 nm or even several µm) raised from the plasma membrane and relatively small exosomes (30–200 nm) generated by the endolysosomal degradation pathway and released by the cells after maturation via the RAS-associated binding GTPase (RAB) pathway [5]. In some cases, cancer cells secrete higher amounts of EVs in comparison with non-transformed cells [4,6], although, for example, EVs secretion in HeLa cells is inhibited by the human papillomavirus (HPV) E6/E7 oncogenes. Thus, EVs secretion in cancer cells is tightly regulated [7].

EVs, which cancer cells release among other factors remodeling the cell microenvironment [8,9]), can transfer between the cells tumor-promoting microRNAs (miRNAs), mRNA, and even proteins [10,11,12,13]. Moreover, cancer EVs inhibit tumor immunosurveillance [10,11], stimulate angiogenesis [14], mediate drug resistance of tumor cells [15,16], and promote malignant transformation of neighboring epithelial cells [17] and fibroblasts [18]. Melanoma EVs can stimulate melanoma cell growth by regulation of gene transcription by non-coding RNA and activation of the PI3K/AKT/mTOR pathway [19,20]. Moreover, melanoma EVs can enter the bloodstream and transform distant cells forming a new metastatic niche [21]. Melanoma EVs also accelerate the migration of melanoma cells to the lung and lymph nodes by activation of the calgranulin A/serum amyloid A/Toll-like receptor4/NFκB signaling cascade and expression of tumor necrosis factor-alpha (TNF-α), vascular endothelial growth factor (VEGF), and hypoxia-inducible factor 1 (HIF-1) [22,23,24]. Taken together, these data point to melanoma EVs as key players in melanoma progression, which contribute to tumor growth, metastasis, and malignancy. 

Fibroblasts, immune cells, and keratinocytes regulate melanocyte physiology and control proliferation, invasion, and angiogenesis of melanoma [1,25,26]. For example, under physiological conditions, keratinocytes control melanocyte proliferation, differentiation, and pigmentation by secretion of paracrine growth factors and intercellular communication via cell adhesion molecules, such as the basic fibroblast growth factor (bFGF) and human growth factor (HGF) [26], although, under stressful conditions such as photodamage, keratinocytes can be a source of pro-inflammatory and growth-promoting factors [27,28]. Malignant transformation of melanocytes is accompanied by a loss of keratinocyte-mediated growth control due to altered expression of cell adhesion molecules [1]. A decrease in the expression of E-cadherin in transformed melanocytes reduces the attachment of transformed melanocytes to the basement membrane that together with upregulation of N-cadherin and the PI3K/AKT/mTOR pathway and activation of pro-migratory protein SNAI1 stimulate melanoma cell migration [29]. 

Melanoma growth is accompanied by acidification of the tumor microenvironment to pH ~6.5 instead of ~7.4 in normal conditions [30]. Acidification of the microenvironment is a significant factor that promotes tumor progression [31] and determines the properties of melanoma EVs [32]. EVs can stimulate migration and invasion of metastatic non-invasive melanoma cells [32]; however, the EVs’ influence on keratinocytes homeostasis was not studied yet.

Here, we found that EVs derived from the culture media of metastatic melanoma cells cultivated at pH 6.5 (“acidified” EVs) demonstrate a higher content of the factors involved in melanoma progression (miR-221, pro-migratory protein SNAI1, stemness marker CD133, and mRNAs coding EGFR, VEGF-α, α3- and V-integrins, and HSP60) than EVs derived from the culture media of the same cells cultivated at pH 7.4 (“normal” EVs). Incubation with “acidified” EVs enhanced the proliferation and migration of metastatic melanoma cells and normal keratinocytes and changed the morphology of the latter to a stem-like phenotype. Moreover, increased activity of the pro-oncogenic signaling pathways and decreased secretion of the cytokines and adhesion factors controlling melanoma growth and migration were observed in keratinocytes upon incubation with both types of EVs. Correlation between diminished expression of the secreted factors with a worse survival prognosis of patients with metastatic melanoma was confirmed by bioinformatic analysis. Thus, acidification of the melanoma environment affects not only the composition and tumor-promoting activity of metastatic melanoma EVs but can also stimulate the transformation of neighboring keratinocytes and the formation of a permissive tumor microenvironment.

## 2. Materials and Methods

### 2.1. Cell Cultivation

Metastatic skin melanoma mel P, mel H, and mel Kor cells were obtained from the patients of the Federal State Budgetary Institute “N.N. Blokhin National Medical Research Center of Oncology” of the Ministry of Health of Russia (Moscow, Russia) after the informed consent and characterized previously [33,34]. The cells were deposited in the Russian Vertebrate cell culture collection (#688D, 715D, and 687D, respectively, St-Petersburg, Russia), where they were taken from. Mel P, mel H, and mel Kor cells were grown in RPMI-1640 media (PanEco, Moscow, Russia) supplemented with 10% FCS (Cytiva, Buckinghamshire, UK) and 1% penicillin/streptomycin (PanEco).

Human immortalized oral Het-1A keratinocytes (CRL-2692, ATCC, Manassas, VA, USA)—used here as a model of normal keratinocytes—were cultivated in the bronchial epithelium basal medium (BEBM, CC-3171, Lonza, Bazel, Switzerland) with the growth factors and supplements (CC-4175, Lonza, Bazel, Switzerland). Before the cell subculturing and performing experiments, culture flasks and plates were pre-coated with a mixture of 0.01 g/L fibronectin (Sigma-Aldrich, St Louis, MO, USA), 0.03 g/L bovine collagen type I (Sigma-Aldrich), and 0.01 g/L bovine serum albumin (Sigma-Aldrich) dissolved in the corresponding culture medium. Cells were maintained at 37 °C in a humidified atmosphere with 8% CO_2_. All types of the cells were subcultured twice per week. The cells were passaged no more than 40 times and regularly tested for the absence of mycoplasma contamination by the PCR kit (Mycoreport, Evrogen, Moscow, Russia).

### 2.2. Melanoma EVs Isolation

EVs derived from the metastatic skin melanoma mel P, mel H, and mel Kor cells were isolated as described previously [35] with some changes. Briefly, cells were seeded in a 25 cm^2^ flask (5 × 10^5^ cells/flask) and grown for 48 h. Then, the media were collected and centrifuged at 10,000× *g* for 15 min (4 °C). After that, the supernatant was centrifuged at 120,000× *g* for 70 min (4 °C). After centrifugation, the protein complexes from fetal calf serum (FCS) contained in the cell media were removed by the exclusion chromatography. For this, EVs were resuspended in BEBM and incubated with the resin Superdex G-250 (GE Healthcare, Chicago, IL, USA) for 1 h at RT. Then, the resin was sedimented, the flowthrough was filtered through a 0.2 µm pore PVDF syringe filter (Millipore, Burlington, MA, USA), and the protein concentration of the filtered supernatant containing isolated EVs was measured using the BCA assay (Sigma-Aldrich, St. Louis, MO, USA). The total protein concentration of the “empty” FCS-supplemented media was subtracted as a background. Isolated EVs were stored for further analysis and experiments, frozen in liquid nitrogen.

### 2.3. Melanoma EVs Characterization

The size of EVs was estimated by DLS using DynaPro Titan instrument (Wyatt Technology, Santa-Barbara, CA, USA) equipped by temperature-controlled MicroSampler. Measurements were performed at 25 °C at the laser wavelength 828 nm and 90° scattering angle. The sample (20 μL) was placed in a microcuvette and waited for the establishment of temperature equilibrium before measurement.

For EVs visualization, cryo-EM was used. The Lacey support carbon films were glow-discharged with PELCO easiGlow (Ted Pella, Redding, CA, USA) at 25 mA plasma current for 30 s, and 3 µL of the EVs sample was applied onto the grids. Then, samples were rapidly plunge-frozen in liquid ethane using Vitrobot Mark IV (Thermo Fisher Scientific, Waltham, MA, USA) after blotting for 1.5 s using a filter paper. Vitrified samples were examined under the Titan Krios 60–300 cryogenic transmission electron microscope (Thermo Fisher Scientific) at 300 kV in low dose mode using EPU software.

To confirm that EVs are membrane-coated particles, they were simultaneously stained by the rabbit antibody for the exosome marker TSG101 (1:100, ABIN2780037, Antibodies Online, Aachen, Germany) and by the secondary AlexaFluor488-conjugated anti-rabbit antibody (1:500, 611-545-215, Jackson Immunoresearch, Cambridge, UK) for 3 h at room temperature and analyzed using the Attune NxT flow cytometer (Life Technologies, Waltham, CA, USA). All buffers were double-filtered through a 0.2 µm PVDF filter (Millipore). EVs stained only by the secondary antibody were used as a negative control. The data were presented as median fluorescence intensities (MFI) normalized to the negative control. Examples of the dot-plots of EVs are shown in Figure S1; at least 100,000 EVs in the gate per sample were analyzed. EVs incubation during 30 min with the 5% detergent Triton X-100 (Applichem, Darmstadt, Germany) led to the disappearance of the spot corresponding to EVs, proving that EVs were membrane-coated structures as described earlier [36].

Expression of the exosomal markers CD63 and TSG101 in EVs was confirmed by Western blotting (Supplementary Figure S2g,h).

### 2.4. Real-Time PCR for mRNA Detection

Total RNA was isolated using the Bio-Rad Aurum RNA mini-isolation kit (Bio-Rad, Hercules, CA, USA) according to the manufacturer’s instructions. cDNA was synthesized by the Mint reverse transcriptase kit and oligodT primer (Evrogen). After that, qPCR was performed with ready to use SYBR Green HS mix (Evrogen) and the primers specific to the *EGFR*, *PDGFRA*, *TNFA*, *BDNF*, *VEGFA*, *ITGA2*, *ITGA3*, *ITGV*, *HSP60*, and *KLF4* genes (Supplementary Table S1); coding EGFR; platelet-derived growth factor receptor alpha (PDGFRα); tumor necrosis factor-alpha (TNF-α); brain-derived neurotrophic factor (BDNF); vascular endothelial growth factor A (VEGFA); integrin subunits alpha 2, 3, 5, and V; heat shock protein 60 (HSP60); and Krüppel-like factor 4 (KLF4). Negative controls contained all the components of the PCR mixture with cDNA replaced by mRNA gave no signal. PCR reactions were carried out using the Roche LightCycler 96 amplifier (Roche, Basel Switzerland). The mRNA expression level was normalized to the S18 ribosomal RNA level for EVs composition analysis and to the S18 and RPL13a genes expression to study the EVs’ influence on the KLF4 expression using the LightCycler SW software (Roche).

### 2.5. Real-Time PCR for miRNA Detection

Total mRNA from EVs was extracted by the Aurum Total RNA Mini Kit (Bio-Rad) according to the manufacturer’s instructions. Total cDNA was synthesized using the Mint reverse transcriptase kit (Evrogen) with miRNA-specific stem-loop primer (Supplementary Table S2). After that, real-time PCR was performed using ready-to-use qPCR mix with the SYBR Green I fluorescent dye (Evrogen) and the primers for detection of miRNA-7, miRNA-21, miRNA-31, miRNA-96, miRNA-135b, miRNA-203, miRNA-221, and miRNA451 described in Table S1. Negative controls contained all the components of the PCR mixture with cDNA replaced by mRNA gave no signal. All PCR reactions were performed using the Roche Light cycler 96 real-time detection thermal cycler (Roche). Data were analyzed by the ΔCt method [37] using the Light-Cycler 96 SW1.01 software (Roche). The expression level of the genes was normalized to the expression level of the housekeeping non-coding RNA U6.

### 2.6. Western Blotting

For immunochemical characterization, EVs derived from the metastatic melanoma cells cultivated in normal and acidic conditions were isolated as described previously. EVs isolated from the FCS-supplemented media were used as the negative control. After that, the total protein concentration was measured by the BCA assay, and EVs were resuspended in the PAGE loading buffer (120 mM Tris-HCl, 20% (*v*/*v*) glycerol, 10% (*v*/*v*) mercaptoethanol, 4% (*w*/*v*) sodium dodecyl sulfate, and 0.05% (*w*/*v*) bromophenol blue, pH 6.8). EVs were submitted to the gel electrophoresis (10 µg of the total protein per the lane), blotted onto nitrocellulose membranes (GE Healthcare), and blocked for 2 h in 5% skim milk (Sigma-Aldrich) in TBS buffer (50 mM Tris, 150 mM NaCl, pH 7.4) containing 0.1% Tween-20 (Applichem, Darmstadt, Germany). The membranes were incubated overnight at 4 °C with the primary antibody against TSG101 (rabbit, 1:1000, ABIN2780037, Antibodies-Online, Aachen, Germany), or CD63 (rabbit, ab217345, 1:1000, Abcam, Cambridge, UK), or cytochrome C (mouse, 1:1000, ab13575, Abcam), washed 3 times with TBS + 0.1% Tween-20 and incubated with the HRP-conjugated secondary anti-rabbit antibody (1:5000, 111-035-003, Jackson Immunoresearch) or anti-mouse antibody (1:5000, 715-035-150, Jackson Immunoresearch) for 1 h at 20 °C. After that, membranes were washed 4 times with TBS + 0.1% Tween-20, and the HRP signal was detected by the ECL substrate (Bio-Rad, Hercules, CA, USA) using the ImageQuant LAS 500 chemidocumenter (GE Healthcare).

For analysis of the EVs influence on the expression of different melanoma progression markers, Het-1A keratinocytes were seeded in 6-well culture plates (20 × 10^5^ cells/well, see later for details), grown for 24 h, and then incubated with mel P-derived EVs for 48 h. After that, the cells were detached by Versene and lysed in the RIPA buffer supplemented with the SIGMAFAST protease inhibitor cocktail (Sigma-Aldrich). The cell content was analyzed by Western blotting. For EGFR detection, the loading buffer contained only 120 mM Tris-HCl, 20% (*v*/*v*) glycerol, pH 6.8; for other cases, the PAGE loading buffer was used. The membranes were incubated overnight at 4 °C with primary mouse anti-EGFR (1:500, sc-120, Santa Cruz, Dallas, TX, USA), or anti-CD44 (1:500, ABIN969026, Antibodies-Online), or anti-CD133 (1:500, ABIN6559815, Antibodies-Online), or rabbit anti-SNAI1 (1:500, NBP2-27293, Novus Bio, Centennial, CO, USA), or rabbit anti-KLF4 (1:2000, NBP2-24749SS, Novus Bio) antibodies, washed 3 times with TBS + 0.1% Tween-20, and incubated with the HRP-conjugated secondary anti-mouse antibody (1:5000, 715-035-150, Jackson Immunoresearch) or anti-rabbit antibody (1:5000, 111-035-003, Jackson Immunoresearch) for 1 h at 20 °C. After that, membranes were washed 4 times with TBS + 0.1% Tween-20, and the HRP signal was detected by the ECL substrate (Bio-Rad, Hercules, CA, USA) using the ImageQuant LAS 500 chemidocumenter (GE Healthcare).

### 2.7. Influence of EVs on Cell Proliferation

For acidification, the cell media of metastatic melanoma cells were supplemented with 25 mM HEPES, pH 6.5. The chosen pH value for the acidic media corresponds to the pH value of melanoma lesions [30] and was not toxic to metastatic melanoma cells. 

To study the influence of EVs derived from the metastatic melanoma cells on mel P and Het-1A proliferation, the cells were seeded in 96-well cell culture plates (5 × 10^3^ cells/well) and grown for 24 h. Thereafter, EVs were dissolved in BEBM, added to the cells at the total exosomal protein concentration of 50 μg/mL, and mel P and Het-1A cells were additionally incubated for 72 h and 48 h, respectively, without the media change. The used total exosomal protein concentration was chosen in accordance with the total protein concentration in exosomes derived from cancer patients’ blood (20–100 μg/mL) [35].

For cell viability analysis, the WST-1 colorimetric test was used [38]. Briefly, WST-1 (water-soluble tetrazolium salt 1; Santa Cruz) and 1-m-PMS (1-methoxy-5-methylphenazinium methyl sulfate, Santa Cruz) were added to the cells in concentrations of 0.25 mM and 5 μM, respectively, for 1 h. Formation of the colored product was measured at 450 nm with background subtraction at 655 nm using the microplate reader Bio-Rad 680 (Bio-Rad, Hercules, CA, USA). The data were normalized to averaged read-out from the control wells, containing cells without added compounds. 

For investigation of the activation of the PI3K and ERK pathways in Het-1A cells upon incubation with EVs, the inhibitors of these cascades Wortmannin (Sigma-Aldrich) and PD98059 (Tocris, Brislot, UK) were used, respectively. Cells were seeded as described above and incubated with 50 µg/mL of EVs in the presence of 15 nM of Wortmannin or 5 µM of PD98059 or a mixture thereof for 48 h.

For additional analysis of the EVs’ influence on keratinocytes proliferation, the BrdU assay was carried out. Het-1A cells were seeded at 6-well culture plates (20 × 10^5^ cells/well), grown for 24 h, and then, mel P-derived EVs (50 µg/mL) were added to the cells. Simultaneously with EVs, the BrdU reagent (10 µM in each well) from the water 10 mM stock was added to the wells, and the cells were incubated for additional 48 h. After that, cells were detached by Versene, fixed in 4% paraformaldehyde for 30 min at RT, permeabilized by 1% Triton X100 for 5 min at RT, and incubated for 2 h with the FITC conjugated anti-BrdU antibody (1:100, MAB3262F, Sigma-Aldrich). Then, the cells were washed by PBS and analyzed using the Attune NxT flow cytometer (Life Technologies). The data were analyzed using the Attune NxT software (Life Technologies) and presented as % of cells, which bound BrdU.

To investigate the influence of EVs derived from mel P cells on the Het-1A cell morphology, the keratinocytes were seeded in 6-well plates (13 × 10^5^ cells/well), grown for 24 h, and incubated with EVs for additional 48 h. After that, plates were imaged at 50× and 30× magnification using the E-scope mode of the CloneSelect Imager (Molecular Devices, San Jose, CA, USA). The 6 photos of each well were taken, and the keratinocytes with a stem-like morphology were counted manually.

### 2.8. Wound Healing (Scratch) and Invasion Assays

The wound healing (scratch) assay was performed as described elsewhere [39] with some changes. In brief, cells were seeded in 96-well cell culture plates (6 × 10^4^ cells/well) and grown for 24 h. After 8 h, the wells were scratched with a sterile 10 μL pipette tip. Then, the cells were washed with PBS and incubated with EVs for additional 24 h (for mel P cells) or 48 h (for Het-1A cells). Pictures were analyzed after 0, 24, and 48 h at 20× magnification at CloneSelect Imager (Molecular Devices). The center of the plate was marked as the central reference point to ensure recording of the same area during the time course. Digital images were taken, and the scratch area was quantified using the ImageJ (NIH, Bethesda, MD, USA) and MS Excel (Microsoft, Redmond, WA, USA) software by the percentage measurement of the scratch surface occupied by the migrating cells. In each experiment, the duplicate measurements were averaged. 

For investigation of the Het-1A cells invasion, the Abcam migration/chemotaxis assay kit (ab235694, Abcam, Cambridge, UK) based on cell migration through the 8 µm pored membrane was used. Cells were seeded in the migration chambers in 24-well plates (2 × 10^5^ cells per well), incubated with the normal or acidic EVs for 48 h, and quantified according to the manufacturer’s protocol.

### 2.9. Flow Cytometry

For the EVs composition analysis, EVs derived from normal and acidified mel P cells were isolated and stained for TSG101 as described in 2.1. After that, EVs were simultaneously incubated with the primary mouse anti-EGFR (1:500, sc-120, Santa Cruz), anti-PDGFR (1:500, ABIN5611263, Antibodies-Online), anti-CD133 (1:500, ABIN6559815, Antibodies-Online), and anti-SNAI1 (1:500, Novus Bio, Centennial, CO, USA) antibodies and the secondary TRITC-conjugated anti-mouse (1:500, 115-025-062, Jackson Immunoresearch) antibodies for 3 h. After that, the EVs samples were diluted 10-fold and analyzed using the Attune NxT flow cytometer (Life Technologies). All buffers were double-filtered through a 0.2 µm PVDF filter (Millipore). Only TSG101-positive EVs were taken for the protein composition analysis. EVs stained only by the secondary antibodies were used as the negative control. At least 100,000 EVs in the gate per sample were analyzed. The data were analyzed using the Attune NxT software (Life Technologies) and presented as MFI, normalized to the negative control.

For analysis of the expression of prooncogenic markers in Het-1A keratinocytes, the keratinocytes were seeded at 6-well plates (13 × 10^5^ cells/well) and grown for 24 h, treated by mel P derived EVs (50 µg/mL) for 48 h, then the cells were fixed for 1 h in 4% paraformaldehyde (Applichem), blocked for 30 min in 5% BSA (Sigma-Aldrich), and stained for 1 h with the primary mouse anti-EGFR (1:500, sc-120, Santa Cruz), anti-PDGFR (1:500, ABIN5611263, Antibodies-Online), anti-CD133 (1:500, ABIN6559815, Antibodies-Online), anti-CD44 (1:500, ABIN, Antibodies-Online), and anti-SNAI1 (1:500, Novus Bio, Centennial, CO, USA) antibodies for 2 h at RT, washed 3 times in PBS and stained with the secondary TRITC-conjugated anti-mouse (1:500, 115-025-062, Jackson Immunoresearch) antibodies for 1 h, washed 2 times and analyzed using the Attune NxT flow cytometer (Life Technologies). Cells stained only with the secondary antibodies were used as the negative control. The gating strategy is shown in Figure S3. The data were analyzed using the Attune NxT software (Life Technologies) and presented as MFI, normalized to the negative control.

### 2.10. Analysis of Intracellular Pathways Activity Using Bio-Plex Magnetic Assay

To analyze the influence of EVs on the phosphorylation of EGFR (Y1173), extracellular-regulated kinase (ERK)1/2 (T202/Y204, T185/Y187), p38 MAP kinase (Thr180/Tyr182), c-Jun N-terminal kinase (JNK)1/2 (T183/Y185), phosphatase and tensin homolog deleted on chromosome 10 PTEN (S380), protein kinase B AKT (S473), and mammalian target of rapamycin mTOR (S2448), the Bio-Plex magnetic assay with the Bio-Plex Pro cell signaling reagent kit (Bio-Rad) was performed. Het-1A cells were seeded in the 6-well plates (13 × 10^5^ cells/well), incubated with EVs derived from normal and acidified mel P cells (50 µg/mL) for 48 h, and lyzed in the provided lysis buffer. Magnetic beads with the covalently attached antibodies were sequentially incubated with the cell lysates (200 µg/mL, 10 µg of the total protein per sample) overnight (30 °C), washed 3 times with the provided wash buffer and magnetic manifold (Bio-Rad), incubated with the detection antibodies for 2 h (30 °C), washed 3 times, incubated with streptavidin-phycoeritrin (PE) for 30 min, washed 3 times, and analyzed using the Attune NxT flow cytometer (Life Technologies). Magnetic beads incubated in the lysis buffer, processed as described, and stained only by streptavidin-PE were used as the negative control. Gating strategies are shown in Figure S4; at least 50 beads in the gate per the sample were analyzed. The data were analyzed using the Attune NxT software (Life Technologies) and presented as MFI, normalized to the negative control. The control lysates of the following cells were used to establish the assay linearity: EGF-treated HeLa cells (EGFR (Y1173), ERK1/2 (T202/Y204, T185/Y187), Akt (S473)); UV-treated HEK293 cells (JNK1/2 (T183/Y185) and p38 MAPK (Thr180/Tyr182); PDGF-treated NH3T3 cells (PTEN (S380), mTOR (S2448)). Beads were incubated with serial dilutions of the lysates. Linearity analysis is presented in Figure S5.

### 2.11. EGFR Knockdown

To block the expression of native EGFR, Het-1A cells were transfected with siRNA (Supplementary Table S3, Synthol, Russia). Cells were seeded in 6-well culture plates (2 × 10^5^ cells/well) and grown for 24 h. Then, 4 different siRNA were mixed (1 μg per well), the mixture was diluted in the 100 μL of the transfection buffer (Pan-Biotech, Aidenbach, Germany), incubated for 5 min, and mixed with 15 μL of the pre-diluted PanFect A-plus transfection reagent (Pan-Biotech). The final mixture was incubated for 30 min and added to Het-1A cells. The cells were incubated in CO_2_ incubator for 4 h, and the cell media were replaced by the fresh one. After 96 h incubation, the cells were detached by Versene and divided into two parts. The first part was incubated with the anti-EGFR mouse primary antibody (sc-373746, Santa-Cruz), washed, and incubated with the secondary TRITC-conjugated antibody (615-025-214, Jackson Immunoresearch). The expression of the surface receptors was analyzed by flow cytometry. The second part of the cells was seeded in 96-well culture plates (5 × 10^4^ cells per well), incubated with EVs (50 µg/mL) for 48 h, and the WST-1 assay was performed as described above.

### 2.12. Analysis of Cytokine Release by Keratinocytes

Het1-A cells were seeded at 6-well plates (13 × 10^5^ cells/well), grown for 24 h, treated by EVs derived from the mel P cells (50 µg/mL) for 48 h; then, the cell media were collected and stored for further analysis at +4 °C for no more than one week. For investigation of the level of soluble E-selectin (sE-selectin), soluble intercellular adhesion molecules 1 and 3 (sICAM-1 and sICAM-3), soluble platelet and endothelial cell adhesion molecule 1 (sPECAM-1), soluble P-selectin, and soluble vascular cell adhesion molecule 1 (sVCAM-1), the bead-based multiplex Flow Cytomix kit was used (BMS812FF, eBioscience, Santa Clara, CA, USA). For detection of tissue plasminogen activator (t-PA), monocyte chemoattractant protein 1 (MCP-1), interleukins 6 and 8 (IL6 and IL8), and soluble tumor necrosis factor ligand superfamily member 5 (sCD40L), the bead-based multiplex cardiovascular 7-plex kit was used (BMS811FF, eBioscience). For interleukins 5, 10, and 12 (IL5, IL10, and IL12), granulocyte-macrophage colony-stimulating factor (GM-CSF), and TNF-related apoptosis-inducing ligand (TRAIL), the ELISA kits were used (KHC0051, Invitrogen, Waltham, MA, USA; BMS215/2, eBioscience; KHC0121, Invitrogen; KHC2031, Invitrogen; BMS2004, eBioscience, respectively). The detection was carried out according to the manufacturers’ instructions; data were normalized to the averaged read-out from the control flow cytometry tubes/ELISA wells, containing only the detection antibodies and streptavidin-PE/HRP conjugates. All cytokines’ concentrations are presented as pg/mL or ng/mL. The ELISA and FlowCytomix data linearity curves are presented in Figure S6.

### 2.13. TCGA Database Analysis

TCGA GTEX (healthy skin biopsies) and SKCM (metastatic melanoma) studies were accessed via the USCS Xena platform [40]. The genes *IL6*, *CXCL8*, *IL10*, *IL12A*, *IL12B*, *VCAM1*, *ICAM1*, *ICAM3*, *PECAM1*, *SELE*, *SELP*, *CCL2*, *PLAT*, *CD40LG*, *CSF2*, and *TNSF10*, coding IL6, IL8, IL10, IL12A, IL12B, VCAM1, ICAM1, ICAM3, PECAM1, SELE, SELP, CCL2, PLAT, CD40LG, CSF2, and TNSF10 proteins, respectively, were selected for the analysis. IL5 mRNA was not found in the metastatic melanoma samples. Patients with non-glabrous primary or metastatic melanoma were subdivided into the two groups with the mRNA level above or below the median value. Overall survival curves were plotted according to the Kaplan–Meier method and compared using the log-rank test directly in the USCS Xena platform interface.

### 2.14. Statistical Analysis

Data are presented as mean ± SEM. Sample numbers (n) are indicated in the figure legends. No exclusion criteria were applied for the experimental data. The data were analyzed using the one-way ANOVA with appropriate multiple comparisons post hoc test, one-sample *t*-test, or two-tailed *t*-test as indicated in the figure legends. The individual experimental points are shown as white dots. For correlation of patients’ survival with the expression of different cytokines and adhesion factors, the log-rank test was used. Differences in the data were considered statistically significant if *p* < 0.05. Analysis was performed using the GraphPad Prism 9.2 software (GraphPad Software, San Diego, CA, USA).

## 3. Results

### 3.1. Acidification of Metastatic Melanoma Environment Makes EVs More Prooncogenic

EVs of melanoma cells can transfer different mRNAs, miRNAs, and proteins [41]. We isolated EVs from the culture media of different metastatic melanoma cell lines (mel P, mel H, and mel Kor) and showed by DLS that their diameter is about 50–200 nm, which is in accordance with the previously published data [42,43]; they are coated by the lipid membrane and express the exosomal markers CD63 and TSG101 (Supplementary Figures S1 and S2). After that, we analyzed the composition of EVs derived from the mel P cells cultivated under acidic (pH 6.5) and normal (pH 7.4) conditions by real-time PCR, flow cytometry, and Western blotting. We revealed that EVs derived from acidified cells contained *EGFR*, *VEGFA*, *ITGA3*, *ITGV*, and *HSP60* mRNAs (Table 1), which code the surface receptors, secreted factors, and migration messengers implicated in the control of cell growth and migration [26,44,45,46,47]. EVs derived from the mel P cells’ media with pH 7.4 did not contain any of these mRNAs (Table 1). Analysis of the mRNA expression pattern in EVs isolated from the mel H and mel Kor cells revealed that these EVs did not contain *EGFR* mRNA, while only in the case of mel Kor cells, EVs possessed *ITGA2* mRNA (Supplementary Tables S4 and S5). Notably, EVs derived from the mel Kor cells cultivated at pH 7.4 contained *BDNF* and *VEGFA* mRNA (Supplementary Tables S4 and S5).

**Figure 1:**
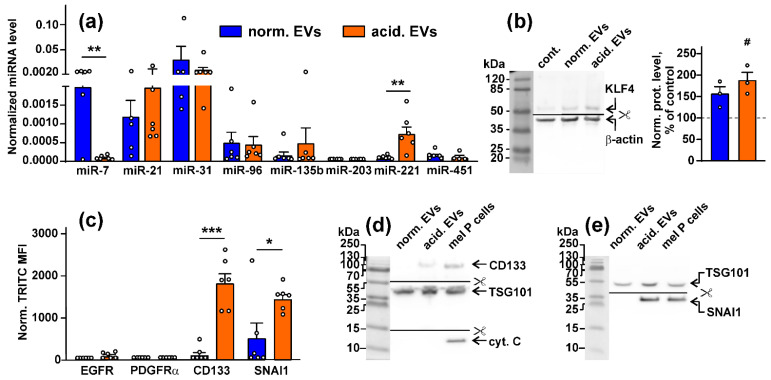
Analysis of the composition of EVs derived from the mel P cells cultivated at pH 6.5 (“acidified”) and pH 7.4 (“normal”): (**a**) expression of different miRNA in “normal” and “acidified” EVs was assayed by real-time PCR with stem-loop primers and normalized to the U6 non-coding RNA. Data presented as the relative miRNA level ± SEM (*n* = 6). ** (*p* < 0.01) indicates significant difference between the data groups according to the two-tailed *t*-test; (**b**) influence of “normal” and “acidified” EVs on expression of KLF4 (miR-7 target) in the Het-1A keratinocytes. Representative Western blot image of KLF4 stained by the specific antibodies in the keratinocytes and the normalized KLF expression level are shown on the left and right panels, respectively. Data presented as a ratio of the KLF expression to the β-actin expression, normalized to the same in the untreated cells ± SEM (*n* = 3). # (*p* < 0.05) indicates significant difference between the treated and untreated cells (control, shown by dashed line) according to the one-sample *t*-test. Whole Western blotting membranes are presented in Figure S9; (**c**) analysis of the EGFR, PDGFRα, CD133, and SNAI1 expression in “normal” and “acidified” EVs by flow cytometry. Data presented as normalized MFI ± SEM (*n* = 6). * (*p* < 0.05) and *** (*p* < 0.001) indicate significant differences between the data groups by the two-tailed *t*-test; (**d**,**e**) analysis of the CD133, SNAI1, TSG101, and cytochrome C expression in EVs by Western blotting. Whole Western blotting membranes are presented in Figure S9.

Analysis of the expression of miRNAs implicated in the melanoma progression [48,49,50,51,52,53,54,55,56] revealed the presence of all miRNAs studied (miRNA-7, miRNA-21, miRNA-31, miRNA-96, miRNA-135b, miRNA-221, and miRNA-451), except miRNA-203 in both types of EVs derived from the mel P cells (Figure 1a). Notably, the expression of anti-melanoma miRNA-7 [57] and miR-221 promoting melanoma progression [56] was significantly suppressed and upregulated, respectively, in “acidified” EVs compared to “normal” EVs (Figure 1a). The similar downregulation of miR-7 and upregulation of miR-221 miRNA were observed for EVs isolated from the mel H and mel Kor cells. It should be noted that the mel H cells did not possess miR-135b (Supplementary Figure S7).

As miRNA can regulate the protein transcription, we analyzed whether miRNA transferred within EVs can modulate the synthesis of its targets in normal keratinocytes. Indeed, the treatment of the Het1-A keratinocytes with “acidic” EVs significantly increased the expression of the miR-7 target—Krüppel-like factor 4 (KLF4)—regulating TGF-β and the WNT-signaling and differentiation in keratinocytes [58], both on the gene and protein levels (Figure 1b, Figures S8 and S9c).

Flow cytometry analysis did not reveal the presence of the EGFR and PDGFRα proteins in both types of EVs derived from the mel P cells, while the increased expression of the stemness marker CD133 and pro-migratory protein SNAI1 in “acidified” EVs was observed (Figure 1c). Elevated expression of the CD133 and SNAI1 proteins in “acidified” EVs was confirmed by Western blotting (Figure 1d,e) that together with the data on the miR-7 and miR-221 miRNA expression (Figure 1a) point on the stronger tumor-promoting properties of “acidified” EVs in comparison to normal ones. We also stained EVs on cytochrome C, which does not express in EVs [59]. Indeed, no cytochrome C was found in both “normal” and “acidified” EVs derived from the mel P cells (Figure 1d).

### 3.2. Metastatic Melanoma EVs Stimulate Growth and Migration of Metastatic Melanoma Cells and Normal Keratinocytes

EVs from the acidified metastatic melanoma cells enhance migration and invasion of the primary non-invasive melanoma cells [32]. Here, we compared the effects of “acidified” and “normal” EVs’ on the growth and migration of the metastatic melanoma cells and normal keratinocytes. Notably, cell growth for 48 h did not change the pH value of the culture media both in the “normal” and “acidified” conditions (Supplementary Figure S10). Control “EVs” isolated from the FCS-supplemented media without addition to cells did not change the viability of the mel P cells (Supplementary Figure S2i).

**Figure 2:**
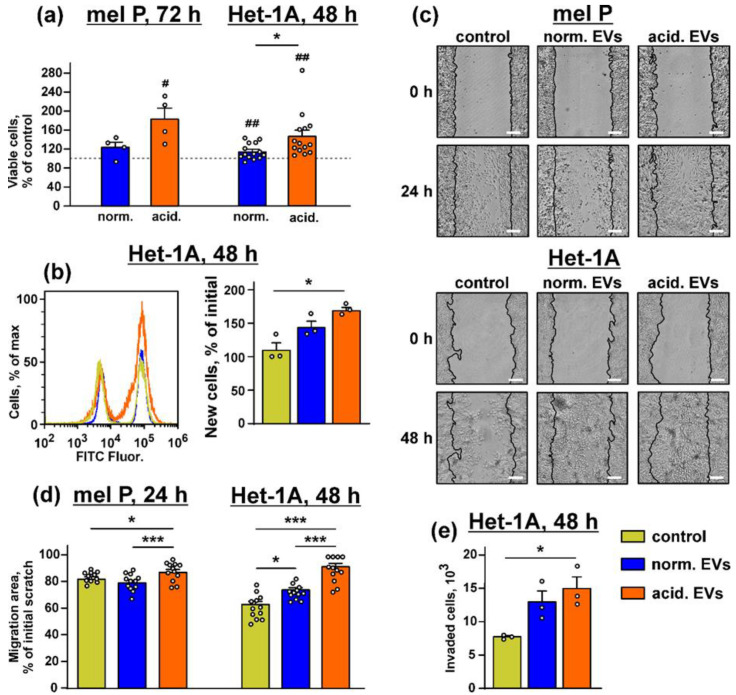
Effect of “normal” and “acidified” EVs on growth and migration of the mel P cells and keratinocytes. (**a**) Influence of “normal” and “acidified” EVs on viability of the mel P and Het-1A cells upon 72 h and 48 h incubation, respectively. Cell viability was assayed by the WST-1 test. Data were normalized to the viability of the untreated cells (control, shown by dashed lines). Data are % of the untreated cells ± SEM (*n* = 4–14), # (*p* < 0.05) and ## (*p* < 0.05) indicate significant difference from the untreated cells by the one-sample *t*-test, and * (*p* < 0.05) indicates significant difference between the data groups by the two-tailed *t*-test; (**b**) analysis of proliferation of the Het-1A cells by the BrdU assay. Data are % of newly divided cells, and * (*p* < 0.05) indicates significant difference between the data groups by the one-way ANOVA followed by Tukey’s post hoc test; (**c**) representative pictures of the scratch test for the mel P and Het-1A cells upon the incubation with “normal” and “acidified” EVs for 24 h and 48 h, respectively; (**d**) scratch square occupied by the migrating mel P and Het-1A cells. Data are presented as % of the scratch surface, occupied by the migrating cells ± SEM (*n* = 12), * (*p* < 0.05) and *** (*p* < 0.001) indicate significant difference between the data groups by the one-way ANOVA followed by Tukey’s post hoc test; (**e**) analysis of invasion of the Het-1A cells through the 8 µm pore chamber. Data presented as the number of cells invaded through the membrane ± SEM (*n* = 3). * *p* < 0.05 indicates significant difference between the data groups by the one-way ANOVA followed by Tukey’s post hoc test.

No significant influence of “normal” EVs derived from the mel P cells on the growth of the mel P and Het-1A cells was revealed, while “acidified” EVs stimulated proliferation of both types of the cells (Figure 2a,b). Similarly, only “acidic” EVs from the mel Kor cells significantly upregulated Het-1A cell proliferation, while no EVs from the mel H cells did it (Supplementary Figure S11a).

Scratch assay showed that “normal” EVs stimulated migration only of the keratinocytes, but the incubation with “acidified” EVs increased migration of both mel P and Het-1A cells (Figure 2c,d). The same effect was observed in the case of the Het-1A cells incubation with EVs isolated from the mel H cells, while the migration enhancement of the keratinocytes was observed upon the incubation with either “normal” or “acidified” EVs derived from the mel Kor cells (Supplementary Figure S11b,c). The invasion assay also showed that “acidified” EVs from the mel P cells significantly increased the number of the Het-1A cells migrated through the 8 µm chamber (Figure 2e).

Thus, “acidified” EVs not only stimulate the migration of the primary melanoma cells [32] but also increase the growth and migration of the normal keratinocytes and metastatic melanoma cells. As the expression of genes and miRNA in EVs derived from the mel P, mel H, and mel Kor cells and the influence of these EVs on growth and migration of the keratinocytes were highly similar, the further work was performed only using EVs isolated from the mel P cells.

### 3.3. Metastatic Melanoma EVs Provide “Stem-like” Morphology of Normal Keratinocytes

**Figure 3:**
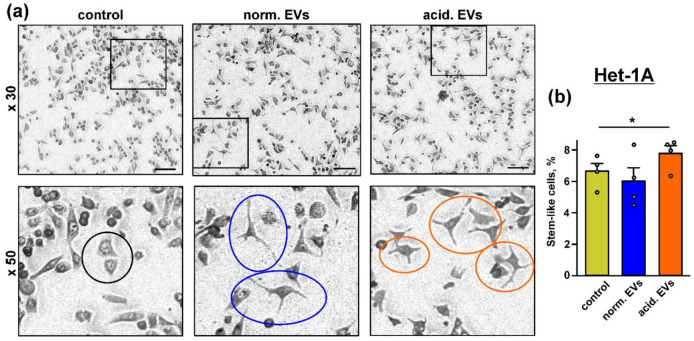
Influence of “normal” and “acidified” EVs on the keratinocytes morphology: (**a**) representative pictures showing the examples of the normal morphology in the untreated Het-1A keratinocytes (black circle) and the “stem”-like morphology in the keratinocytes upon the incubation with “normal” (blue circles) and “acidified” (red circles) EVs. Scale bar 50 µm; (**b**) number of the keratinocytes with the “stem”-like morphology in the untreated cells (control) and after the 48 h incubation with “normal” and “acidified” EVs. Data presented as % of the cells with the “stem”-like morphology ± SEM (*n* = 4); * (*p* < 0.05) indicates significant difference between the data groups by the one-way ANOVA followed by Tukey’s post hoc test.

Acidification drives the changes of the morphology of the primary melanoma cells to the more “stem”-like phenotype [60]. Here, we studied whether “acidified” EVs derived from the mel P cells can change the morphology of the normal keratinocytes. Microscopic observations revealed that the incubation during 48 h with both types of EVs resulted in the changes of the morphology of some keratinocytes from the epithelial-like polygonal shape with regular dimensions to the more mesenchymal, “stem-like” phenotype characterized by the relatively small cell body and the formation of cell “progenitors” (Figure 3a). The number of the cells with the stem-like morphology was significantly higher upon the incubation of the keratinocytes with “acidified” EVs (Figure 3b).

### 3.4. Metastatic Melanoma EVs Activate Intracellular Signaling Pathways and Factors, Which Mediate Growth, Migration, and Stemness in Normal Keratinocytes

**Figure 4:**
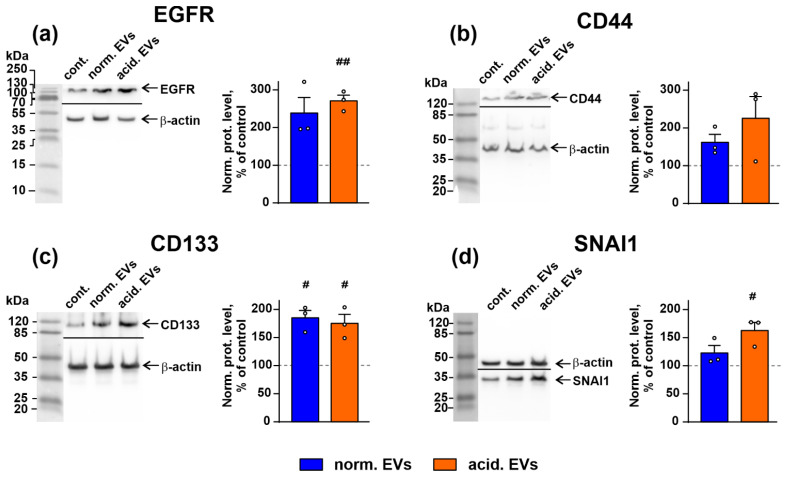
Influence of “normal” and “acidified” EVs on the expression of EGFR (**a**), CD44 (**b**), CD133 (**c**), and SNAI1 (**d**) in the keratinocytes. Representative Western blot images of EGFR, CD133, CD44, and SNAI1 stained by the specific antibodies in the keratinocytes and the normalized expression levels of the corresponding proteins are shown on the left and right panels, respectively. Data presented as the ratio of the expression level of the target protein to the level of β-actin, normalized to the untreated cells ± SEM (*n* = 3). # (*p* < 0.05) and ## (*p* < 0.01) indicate significant differences between the treated and untreated cells (control, shown by dashed lines) according to the one-sample *t*-test. Whole Western blotting membranes are presented in Figure S13.

Changes of the cell morphology observed upon the incubation with metastatic melanoma EVs (Figure 3) reflect the changes in cell homeostasis [61] and can point to the activation of the tumor-promoting secondary messengers in the normal keratinocytes. In line with this, the Western blot analysis revealed the upregulation of the expression of the prooncogenic receptor EGFR, pro-migration factor SNAI1, and stemness marker CD133 in the keratinocytes upon the exposure to “acidified” EVs (Figure 4). Increased expression of CD133 was stimulated by both “normal” and “acidified” EVs (Figure 4c). The expression of another cancer stemness marker—an adhesion receptor CD44—seemed to be upregulated too, but the effect was not statistically significant (Figure 4b). The data on the EGFR, CD44, CD133, and SNAI1 expression were also confirmed by the flow cytometry analysis (Supplementary Figure S12). Notably, the KLF-4 upregulation (Figure 1b) is in line with our observation of induction of the stem-like properties in the keratinocytes by “acidic” EVs. Despite the participation of KLF4 in the differentiation of the keratinocyte [58], it can also upregulate the transcription of many genes, indispensable for EMT and cell growth, so it also serves as an oncogene in many cancers [62,63].

**Figure 5:**
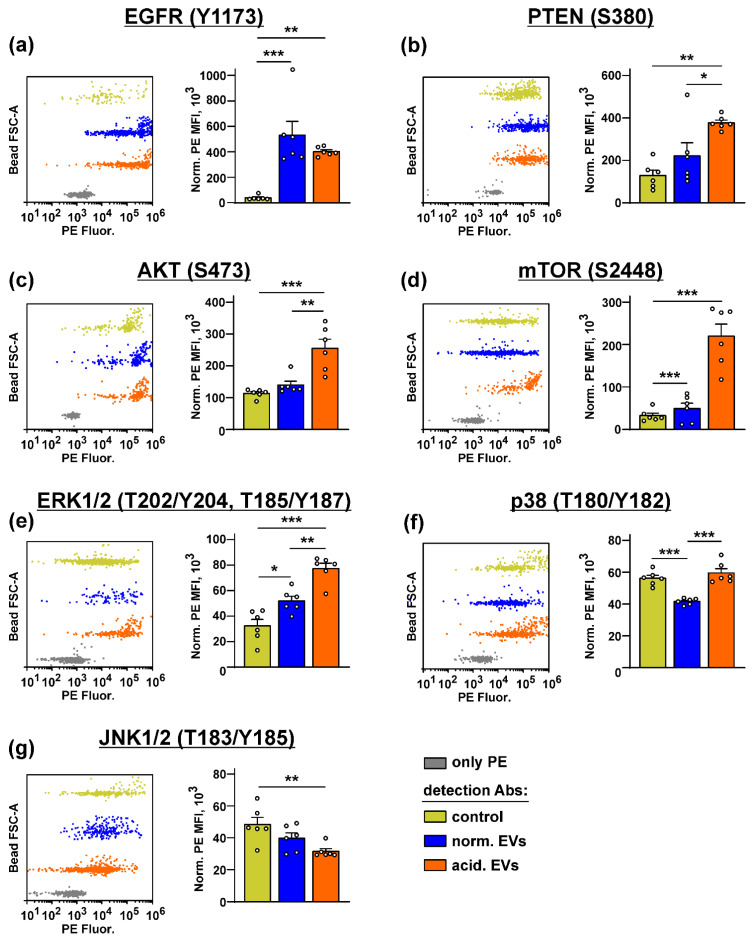
Influence of “normal” and “acidified” EVs on the activity of the different intracellular signaling pathways in the keratinocytes. Het1-A cells were incubated with “normal” and “acidified” EVs for 48 h, and the phosphorylation of EGFR (Y1173) (**a**), PTEN (S380) (**b**), AKT (S473) (**c**), mTOR (S2448) (**d**), ERK1/2 (T202/Y204, T185/Y187) (**e**), p38 MAP kinase (T180/Y182) (**f**), and JNK1/2 (T183/Y185) (**g**) was assayed by the Bio-Plex magnetic beads assay. Representative distributions of the magnetic beads incubated with the lysates of the untreated (control) or treated by EVs’ keratinocytes and the beads stained only by PE are shown on the left panels (every bead probe was analyzed separately and combined on the one panel for illustration). Data on the analysis of the phosphorylation level of the messengers are shown on the right panels. Data were acquired by the Attune NxT flow cytometer and presented as normalized MFI ± SEM (*n* = 6). * (*p* < 0.05), ** (*p* < 0.01), and *** (*p* < 0.001) indicate significant difference between the data groups by the one-way ANOVA followed by Tukey’s post hoc test.

Bio-plex analysis revealed that both types of EVs increased the phosphorylation of EGFR, mTOR, and ERK1/2 in the keratinocytes (Figure 5a,d,e). “Acidified” EVs also activated AKT but inhibited the PTEN and JNK1/2 activity (Figure 5b,c,g). Please note that the increase in the PTEN phosphorylation indicates its inactivation and the loss of the AKT control [64]. The p38 MAPK activity was inhibited by “normal” EVs and restored to the control level by “acidified” EVs (Figure 5f). Thus, the incubation of the keratinocytes with EVs activates the AKT/mTOR and ERK signaling pathways, which regulate the growth and migration of the epithelial cells [65].

**Figure 6:**
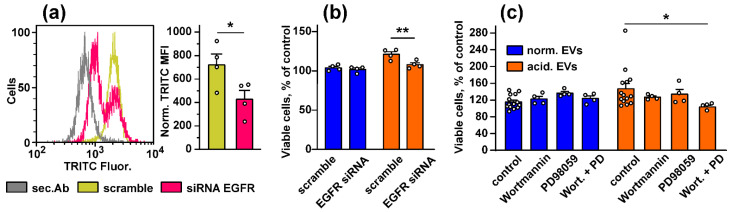
Influence of the EGFR knock-down and inhibition of the PI3K/AKT/mTOR and MEK/ERK pathways on stimulated growth of the keratinocytes induced by EVs. (**a**) Analysis of the EGFR knock-down in the keratinocytes. The representative cell distribution histograms after transfection of the keratinocytes by scramble siRNA and EGFR-specific siRNA and quantification of the EGFR expression are shown on the left and right panels, respectively. Data presented as normalized MFI ± SEM (*n* = 4). * (*p* < 0.05) indicates significant difference between the data groups by the two-tailed *t*-test; (**b**) influence of the EGFR knock-down on viability of the keratinocytes stimulated by “normal” and “acidified” EVs. Data are % of the untreated cells ± SEM (*n* = 4–14), ** (*p* < 0.01) indicates significant difference between the data groups by the two-tailed *t*-test; (**c**) influence of the inhibitors of the PI3K/AKT/mTOR and MEK/ERK pathways (Wortmannin and PD98059, respectively) on the effects of “normal” and “acidified” EVs on viability of the keratinocytes. Control is the cells treated by only EVs (without the inhibitors). Data are % of the untreated cells ± SEM (*n* = 4–14); * (*p* < 0.05) indicates the significant difference between the data groups by the one-way ANOVA followed by Dunnet’s post hoc test.

To prove the EGFR involvement in the changes induced in the keratinocytes upon the treatment with EVs, we knocked out the EGFR expression in the Het-1A cells by siRNA (Figure 6a) and analyzed the EV-induced growth stimulation of the keratinocytes. Indeed, “acidified” EVs did not enhance the proliferation of the Het-1A cells after the EGFR knock-down (Figure 6b). This points to EGFR as the mediator of the EV-induced stimulation of proliferation of the keratinocyte.

To establish the relationship between the upregulation of the PI3K/AKT/mTOR and MEK/ERK pathways and stimulation of growth and migration of the keratinocytes induced by EVs, we used the inhibitors of the PI3K/AKT/mTOR and MEK/ERK pathways (Wortmannin and PD98059, respectively). Inhibitor analysis revealed that both Wortmannin and PD98059 did not affect the proliferation of the keratinocytes, but their combined application completely abolished increased growth of the keratinocytes observed upon the incubation with “acidified” EVs (Figure 6c). “Normal” EVs did not affect the growth of the keratinocytes, and the inhibition of the PI3K/AKT/mTOR and MEK/ERK pathways did not influence on proliferation of the Het1-A cells treated by “normal” exosomes (Figure 6c).

Thus, EVs released by the metastatic melanoma cells stimulate the growth of the normal keratinocytes via the activation of EGFR and the PI3K/AKT/mTOR and MEK/ERK intracellular signaling pathways.

### 3.5. Metastatic Melanoma EVs Inhibit Release of Immunomodulatory Cytokines and Adhesion Factors by Keratinocytes

**Figure 7:**
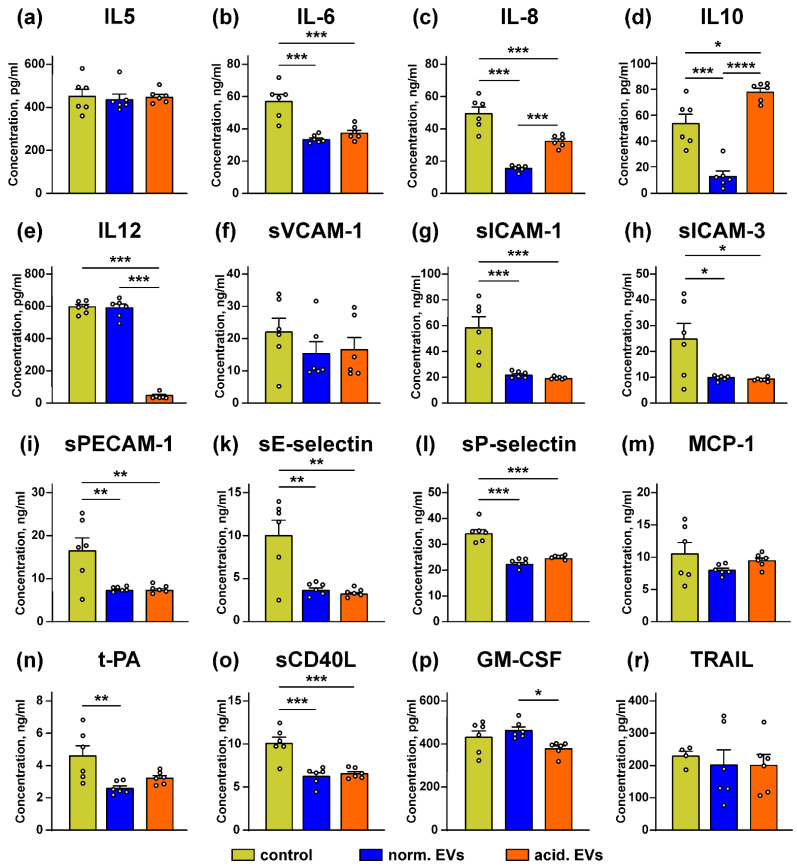
Effect of “normal” and “acidified” EVs on secretion of the different cytokines and adhesion factors by the keratinocytes. Het1-A cells were incubated with “normal” and “acidified” EVs for 48 h, and the concentration of IL5 (**a**), IL10 (**d**), IL12 (**e**), GM-CSF (**p**), and TRAIL (**r**) was assayed by ELISA. Concentration of IL6 (**b**), IL8 (**c**), sVCAM-1 (**f**), sICAM-1 (**g**), sICAM-3 (**h**), sPECAM-1 (**i**), sE-selectin (**k**), sP-selectin (**l**), MCP-1 (**m**), t-PA (**n**), and sCD40L (**o**) was analyzed by the Flow Cytomix kits. Data presented as the normalized protein concentration ± SEM (*n* = 6). Control corresponds to the untreated cells. * (*p* < 0.05), ** (*p* < 0.01), *** (*p* < 0.001), and **** (*p* < 0.0001) indicate significant difference between the data groups by the one-way ANOVA followed by Tukey’s post hoc test.

Keratinocytes control the growth of melanocytes and the melanoma progression by secretion of the different paracrine factors [1,25,26]. We tested whether “normal” and “acidified” EVs affect secretion by the keratinocytes of the anti-inflammatory cytokines and adhesion factors. It was revealed that the incubation with “normal” EVs led to the significant decrease in secretion of the IL6, IL8, and IL10 immunomodulatory cytokines and different cell adhesion regulators, which are also capable of attracting the immunocompetent cells to the tumor lesion: sICAM-1, sICAM-3, sPECAM-1, sE and sP-selectins, t-PA, and sCD40L (Figure 7b–d,g–i,k,l,n,o). “Acidified” EVs inhibited release of the IL6, IL8, IL12 cytokines and the sICAM-1, sICAM-3, sPECAM-1, sE- and sP-selectins, and sCD40L adhesion factors (Figure 7b,c,e,g–i,k,l,o). It should be noted that “acidified” EVs decreased secretion of IL8 significantly weaker than “normal” ones. Additionally, in contrast to “normal” EVs, “acidified” EVs upregulated the secretion of IL10 (Figure 7d). No influence of both types of EVs on secretion of IL5, sVCAM-1, MCP-1, GM-CSF, and TRAIL compared to the untreated cells (control) was observed (Figure 7a,f,m,p,r).

### 3.6. Upregulation of Immunomodulatory Cytokines and Adhesion Factors in Tissues from Patients with Metastatic Melanoma Correlates with Better Survival Prognosis

**Figure 8:**
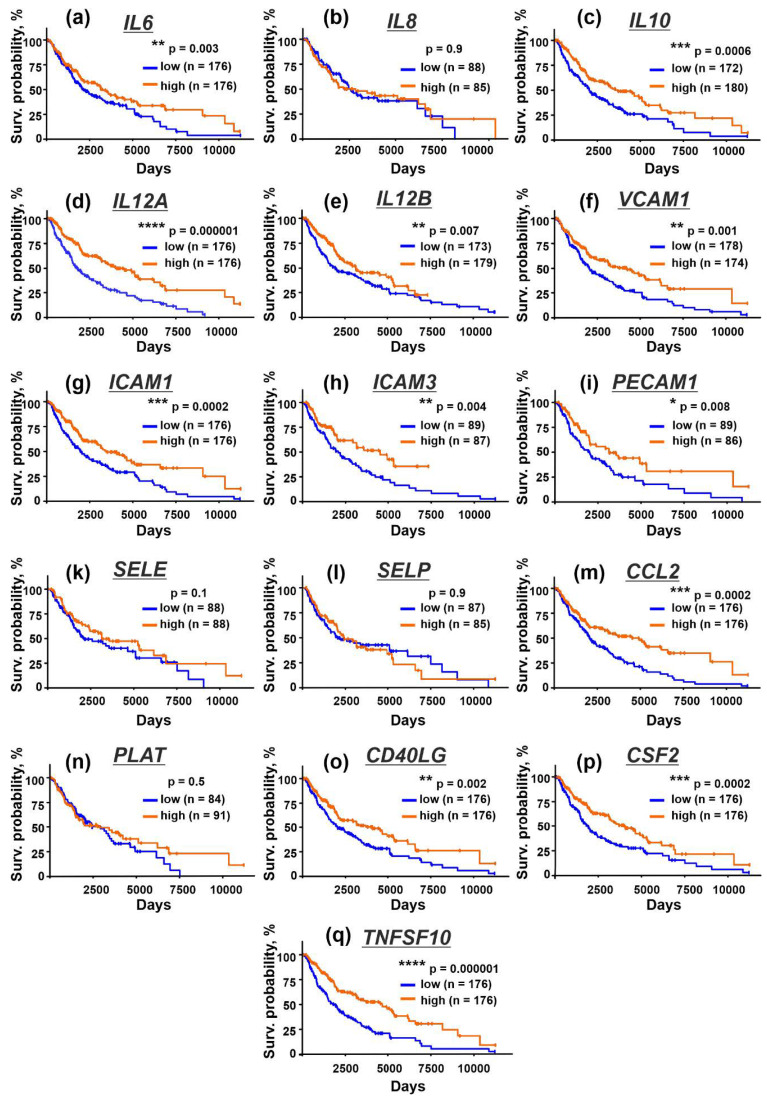
Kaplan–Meier analysis of the correlation between the survival of the patients with metastatic melanoma and the different expression of the genes coding IL6 (**a**), IL8/CXCL8 (**b**), IL10 (**c**), IL12A (**d**), IL12B (**e**), VCAM1 (**f**), ICAM1 (**g**), ICAM3 (**h**), PECAM1 (**i**), SELE (**k**), SELP (**l**), CCL2 (**m**), PLAT (**n**), CD40LG (**o**), CSF2 (**p**), and TNSF10 (**q**). * (*p* <0.05), ** (*p* < 0.01), *** (*p* < 0.001), and **** (*p* < 0.0001) indicate significant difference between the overall survival prognosis for the patients with the high (above median) and low (below median) gene expression according to the log-rank test.

To investigate the clinical relevance of the obtained data, we analyzed the relation between the expression of the immunomodulatory cytokines and adhesion factors and the survival of the patients with metastatic melanoma from the TCGA database (SKCM). Enhanced level of mRNA coding the IL6, IL10, IL12, sICAM-1, sICAM-3, sPECAM-1, and CD40L proteins, whose secretion by the keratinocytes was inhibited by EVs (Figure 7b,d,e,g–i,o), correlated with the better survival prognosis of the patients with metastatic melanoma (Figure 8a,c–e,g–i,o). High level of mRNA coding the sVCAM-1, MCP-1, GM-CSF, and TRAIL proteins, whose secretion was not affected by EVs (Figure 7f,m,p,r), was also associated with the better survival prognosis (Figure 8f,m,p,q). At the same time, the expression of E- and P-selectins downregulated by both types of EVs (Figure 7k,l) did not correlate with the patients’ survival (Figure 8k,l).

Thus, the downregulation of IL6, IL10, IL12, sICAM-1, sICAM-3, sPECAM-1, and CD40L secretion may lead to the melanoma progression and impair survival of the patients with metastatic melanoma.

## 4. Discussion

Keratinocytes control differentiation, pigmentation, and stress repair of the melanocytes and shape the microenvironment during their malignant transformation [1,25,26]. Cross-talk between the melanoma cells and surrounding normal tissue is mediated via the secreted factors and EVs, but the molecular mechanisms underlying the transformation of the neighboring normal keratinocytes and their education for the formation of the permissive tumor microenvironment by the melanoma cells remain poorly studied. 

Here, we performed the comparative analysis of the composition and effects of EVs derived from the metastatic melanoma cells cultivated at the physiological (pH 7.4) and acidified (pH 6.5) conditions on the normal keratinocytes. Besides miRNAs implicated in melanoma growth, migration, and normal cell transformation [48,49,53,54,55,56,57,66] and common with “normal” EVs, “acidified” EVs contained the less amount of miR-7 and a higher amount of miR-221 (Figure 1a). The simultaneous downregulation of miRNA-7 (inhibiting melanoma growth and migration [57]) and upregulation of miR-221 (promoting melanoma progression [56]) are linked with the melanoma progression. Interestingly, miR-203, which was not found in both types of EVs, inhibits melanoma cell growth by the induction of the tumor cell senescence [67]. Moreover, “acidified” EVs demonstrated the increased expression of the stemness marker CD133 [68], migration messenger SNAI1 [69], and EGFR (Table 1, Figure 1d,e) in comparison with “normal” EVs. Notably, the previous proteome analysis of “normal” and “acidified” melanoma EVs revealed many differences in the expression of the prooncogenic proteins [32]; however, the increased expression of SNAI1 and CD133 in “acidified” EVs was revealed here for the first time. Thus, the data obtained indicate that the acidification of the metastatic melanoma environment makes EVs more prooncogenic. 

Notably, the stemness regulator KLF4, which was upregulated in the keratinocytes upon the treatment with “acidic” EVs (Figure 1b), is the target for miR-7 miRNA, which was downregulated in acidic EVs in comparison with “normal” ones (Figure 1a). On the other hand, “acidic” EVs contained the increased amount of *EGFR* mRNA (Table 1), and the increased EGFR expression on the protein level was observed in the keratinocytes treated by “acidic” EVs. We propose that the miRNAs and mRNAs transfer by the exosomes can regulate the stemness and prooncogenic properties of not only the cancer cells but of normal ones too.

In line with this suggestion, EVs from the acidified melanoma cells stimulated the growth and migration of both metastatic melanoma cells and keratinocytes (Figure 2 and Figure S11). These data, together with the data on the appearance of the stem-like morphology of the keratinocytes after the incubation with “acidified” EVs (Figure 3), agree well with the upregulated expression of the EGFR, CD133, SNAI1, and KLF4 proteins in the treated keratinocytes (Figure 1b and Figure 4a,c,d, respectively). EVs from the melanoma cells did not change the expression of CD44, another cancer stem cell marker and EMT inducer; however, this protein is indispensable for proliferation and differentiation of the keratinocytes, so it is strongly activated in this type of cells [70]. 

The study of the phosphorylation of the messengers implicated in the different intracellular signaling cascades in the keratinocytes after the incubation with EVs revealed the increased activity of EGFR, AKT, mTOR, and ERK1/2 and the decreased PTEN activity. Notably, the changes in their activity were more obvious in the case of “acidified” EVs (Figure 5). Thus, the incubation of the keratinocytes with EVs derived from the metastatic melanoma cells activates the AKT/mTOR and ERK signaling pathways, which are implicated in the control of cell migration [65,71]. Inhibition of the p38 MAP kinase phosphorylation by “normal” EVs can indicate the growth suppression of migrating epithelium [72], although the incubation with “acidified” EVs did not affect the phosphorylation of this kinase (Figure 5). Inactivation of JNK, which demonstrates the pro-apoptotic activity [73], may point to the apoptosis inhibition in the keratinocytes upon the incubation with EVs [73]. Using the EGFR knock-down and inhibitors of the PI3K/AKT/mTOR and MEK/ERK signaling pathways, we found that the upregulation of proliferation of the keratinocytes induced by “acidic” EVs is mediated by EGFR and the simultaneous activity of the PI3K/AKT/mTOR and MEK/ERK pathways (Figure 6). Interestingly, the simultaneous activity of the PI3K/AKT/mTOR and MEK/ERK pathways is characteristic for many tumor cells, including melanomas [74]. Thus, the targeting of both these pathways can not only prevent the melanoma progression but also impair the formation of the melanoma permissive microenvironment by the melanoma-surrounding cells. EGFR activation together with the activation of AKT, mTOR, and ERK1/2 may point to the participation of the EGFR/ERK1/2 and EGFR/PI3K/AKT/mTOR pathways, but not of the EGFR/JNK related cascades in the migration enhancement of the keratinocytes like it takes place in the primary ocular epithelium cells [65]. Notably, miR-7 inhibits the EGFR expression and AKT activity in the melanoma [57] and carcinoma [75] cells. Thus, we believe that the decreased expression of miR-7 in EVs may be linked not only with the upregulation of KLF4 (Figure 1a,b) but also with the upregulation of the EGFR-PI3K/AKT/mTOR related pathways in the keratinocytes treated by “acidified” EVs. In line with it, the absence of *EGFR* mRNA in “normal” EVs (Table 1) can explain the more prooncogenic influence of “acidic” EVs on the growth, migration, and stemness of the normal keratinocytes (Figure 2 and Figure 3).

Keratinocytes control the physiology of the melanocytes by secretion of the different paracrine factors [26,27,28]. Here, we showed that EVs from the metastatic melanoma cells inhibit the release by keratinocytes of the IL6, IL8, IL10, and IL12 immunomodulatory cytokines and the regulators of cell adhesion and immune cell infiltration, such as sICAM-1, s-ICAM-3, sPECAM-1, sE- and sP-selectins, t-PA, and sCD40L (Figure 7). All these secreted factors play a crucial role in the antitumor immunity formation. For example, IL8 shapes the antitumor immunity by the attraction of the antitumor neutrophils or pro-tumor myeloid-derived suppressor cells [76]. The attraction of the monocytes, neutrophils, and NK cells to endothelium is mediated by PECAM-1 [77]. sICAM-3 is important for differentiation of the cytotoxic T cells [78], and CD40L is necessary for the activation of the tumor-infiltrating dendritic cells [79]. IL12 activates the cytotoxic T cells and induces the release of interferon-γ to fight the tumor progression [80]. 

It should be noted that the autocrine production of IL10 inhibits the secretion of IL12 by the macrophages, pointing to the regulation of the immunity by the ratio of these cytokines [81]. Moreover, the upregulation of IL10 with the concomitant downregulation of IL12 is characteristic for the immunosuppressive microenvironment [82]; thus, the upregulation of IL10 with the significant downregulation of the IL12 secretion by the keratinocytes may indicate that “acidic” EVs drive the formation of the immunosuppressive microenvironment during the melanoma progression. In addition, the KLF4 overexpression drives the IL-10 transcription in the macrophages [83], so the increase in IL10 secretion upon the incubation of the keratinocytes with “acidic” EVs (Figure 7d) may be the consequence of the KLF4 upregulation in the Het-1A cells.

Inhibition of the t-PA release impairs carcinogenesis at the early stage, but this cytokine is also able to activate the cytotoxic T cells and promote tumor surveillance [84]. IL6 activates the antitumor immunity [85], regulates the secretion of the anti-inflammatory cytokine IL10 [86] in the melanoma cells via the PI3K and ERK related pathways [87], and promotes the E/P-selectin- and ICAM-1-dependent extravasation of the cytotoxic T cells in melanoma lesions in vivo [88]. In line with this, E-selectin, ICAM-1, and VCAM-1 are upregulated on the surface of the activated endothelial cells, which attract the different immune cells to the tumor lesion [89]. Thus, diminished secretion of E/P-selectins and ICAM-1 in the keratinocytes can be related to the inhibition of IL6 secretion observed upon the incubation with metastatic melanoma EVs (Figure 7). Taken together, our data mean that metastatic melanoma EVs can not only stimulate the prooncogenic processes in the normal keratinocytes resulting in their transformation but also cause the prooncogenic modulation of the immunity in the tumor microenvironment by the suppression of secretion of the cytokines and adhesion factors by the keratinocytes. 

In line with this, the bioinformatic analysis of the TCGA database revealed that the high expression of the genes coding IL6, IL10, IL12, ICAM-1, ICAM-3, PECAM-1, and CD40L in tissues of patients with metastatic melanoma correlates with the better survival prognosis (Figure 8). In contrast, the increased plasma concentration of IL6 and IL8 is linked with the worse survival for patients with metastatic melanoma [90]. This contradiction between our data on decreased IL-6 and IL-8 secretion by the keratinocytes treated by melanoma EVs (Figure 7b,c) and the bioinformatic analysis (Figure 8) can be explained by the note that we studied cytokine secretion by the keratinocytes and modeled the local tumor microenvironment. At the same time, plasma IL6 and IL8 are secreted by the macrophages, neutrophils, or mast cells [91]. Additional studies could shed light on the relationship between intercellular and plasma cytokine secretion in cancer.

## 5. Conclusions

We confirmed that metastatic melanoma EVs can transfer the different prooncogenic mRNAs, miRNAs, and protein factors. EVs derived from the acidified metastatic melanoma cells change the morphology of the normal keratinocytes and enhance their growth and migration by the activation of the prooncogenic intracellular cascades. EVs inhibit the release of the cytokines and adhesion factors by the keratinocytes, which can modulate the antitumor immunity in tumor tissues. Correlation between the increased expression of some cytokines in metastatic melanoma tissues with the better survival prognosis for the patients with metastatic melanoma was revealed. Data obtained reflect the “education” of the normal cells from the tumor microenvironment for the formation of the new metastatic niche.

## Figures and Tables

**Table 1:** Analysis of mRNA contained in EVs derived from the mel P cells cultivated at pH 6.5 and pH 7.4. Expression is normalized to the mRNA level ± SEM (*n* = 8).

Gene	“Normal” EVs (pH 7.4)	“Acidic” EVs (pH 6.5)
*EGFR*	-	1.72 × 10^−5^ ± 4.7 × 10^−6^
*PDGFRA*	-	-
*TNFA*	-	-
*BDNF*	-	-
*VEGFA*	-	8.49 × 10^−4^ ± 1.7 × 10^−4^
*ITGA2*	-	-
*ITGA3*	-	2.87 × 10^−3^ ± 6.4 × 10^−4^
*ITGV*	-	1.1 × 10^−5^ ± 5.5 × 10^−6^
*HSP60*	-	2.26 × 10^−6^ ± 1.7 × 10^−7^

## Data Availability

Data generated within experiments are available on request.

## Supplementary Materials

The following are available online at https://www.mdpi.com/article/10.3390/biomedicines10030660/s1, Figure S1: Exosome characterization by flow cytometry, Figure S2: Characterization of EVs derived from mel P, mel H, and mel Kor cells, Figure S3: Gating strategy for analysis of EVs influence on expression of prooncogenic markers in keratinocytes, Figure S4: Gating strategy for analysis by Bio-Plex magnetic bead assay of the intracellular pathways activated in keratinocytes, Figure S5: The Bio-Plex assay linearity analysis, Figure S6: The ELISA’s and Flow Cytomix kits assays linearity analysis, Figure S7: Analysis of miRNA expression in EVs derived from metastatic melanoma cells mel H and mel Kor cultivated at pH 6.5 (“acidified”) and pH 7.4 (“normal”), Figure S8: PCR analysis of the *KLF* expression in keratinocytes treated by “normal” and “acidified” EVs derived from mel P cells. Figure S9: Whole membranes used for CD133, SNAI1, and KLF expression analysis by Western blotting, Figure S10: Media pH changes upon mel P cells cultivation in “normal” and “acidic” media, Figure S11: Effect of “normal” and “acidified” EVs on growth and migration of keratinocytes, Figure S12: Influence of “normal” and “acidified” EVs on expression of EGFR, CD133, CD44, and SNAI1 in keratinocytes, Figure S13: Whole membranes used for analysis of influence of “normal” and “acidified” EVs on expression of EGFR, CD133, CD44, and SNAI1 in keratinocytes, Table S1: Primers, used for qPCR experiments, Table S2: Primers used for analysis of miRNA expression, Table S3: The siRNA sequences, Table S4: Analysis of mRNA contained in EVs derived from the mel H cells cultivated at pH 6.5 and pH 7.4, Table S5: Analysis of mRNA contained in EVs derived from the mel Kor cells cultivated at pH 6.5 and pH 7.4.
