# Supplementary material for: Extracellular Vesicles Derived from Acidified Metastatic Melanoma Cells Stimulate Growth, Migration, and Stemness of Normal Keratinocytes
Source: Biomedicines. 2022 Mar 12;10(3):660. doi: 10.3390/biomedicines10030660 (PMC8945455; doi:10.3390/biomedicines10030660)
Supplement: Supplementary file 1 [file biomedicines-10-00660-s001.zip › biomedicines-1514884-supplementary.pdf]

biomedicines

MDPI

Supplementary material

Extracellular Vesicles Derived from Acidified Metastatic Melanoma Cells Stimulate Growth, Migration, and Stemness of Normal Keratinocytes

Maxim L. Bychkov [1], Artem V. Kirichenko [1,2], Irina N. Mikhaylova [3], Alexander S. Paramonov [1], Evgeny V. Yastremsky [4,5], Mikhail P. Kirpichnikov [1,6], Mikhail A. Shulepko [1] and Ekaterina N. Lyukmanova [1,2,6,*]

1 Shemyakin-Ovchinnikov Institute of Bioorganic Chemistry, Russian Academy of Sciences, 119997 Moscow, Russia; maksim.bychkov@gmail.com (M.L.B.); bittert@mail.ru (A.V.K.); a.s.paramonov@gmail.com (A.S.P.); mikhailshulepko@gmail.com (M.A.S.)
2 Moscow Institute of Physics and Technology, State University, 141701 Dolgoprudny, Russia
3 Federal State Budgetary Institution N.N. Blokhin National Medical Research Center of Oncology, Ministry of Health of Russia, 115548 Moscow, Russia; irmikhaylova@gmail.com
4 National Research Center Kurchatov Institute, Akademika Kurchatova pl. 1, 123182 Moscow, Russia; e.yastremsky@gmail.com
5 Shubnikov Institute of Crystallography of Federal Scientific Research Centre Crystallography and Photonics of Russian Academy of Sciences, Leninskiy Prospect 59, 119333 Moscow, Russia
6 Interdisciplinary Scientific and Educational School of Moscow University «Molecular Technologies of the Living Systems and Synthetic Biology», Faculty of Biology, Lomonosov Moscow State University, 119234, Moscow, Russia
* Correspondence: ekaterina-lyukmanova@yandex.ru

Citation: Bychkov, M.L.; Kirichenko, A.V.; Mikhaylova, I.N.; Paramonov, A.S.; Yastremsky, E.V.; Kirpichnikov, M.P.; Shulepko, M.A.; Lyukmanova, E.N. Extracellular Vesicles Derived from Acidified Metastatic Melanoma Cells Stimulate Growth, Migration, and Stemness of Normal Keratinocytes. *Biomedicines* **2022**, *10*, 660. https://doi.org/10.3390/biomedicines10030660

Academic Editor: Paul Rösch

Received: 2 December 2021
Accepted: 9 March 2022
Published: 12 March 2022

Publisher's Note: MDPI stays neutral with regard to jurisdictional claims in published maps and institutional affiliations.

Copyright: © 2022 by the authors. Licensee MDPI, Basel, Switzerland. This article is an open access article distributed under the terms and conditions of the Creative Commons Attribution (CC BY) license (https://creativecommons.org/licenses/by/4.0/).

Table S1. Primers, used for qPCR experiments.

Gene	Primer		Amplicon size, bp
	Forward	Reverse	
S18 SSU RNA	CTCAACACGGGAAAC-CTCAC	CGCTCCACCAACTAA-GAACG	110
RPL13a	*TCAAAGCCTTCGCTAG-TCTCC*	GGCTCTTTTTGCCCG-TATGC	104
EGFR	GAAATCATACGCGGCAG-GAC	TGAGGGAGCG-TAATCCCAAG	91
PDGFRA	GACATCATGCGGGACTCGAA	CATGGGCAGCTCTGGG-TAAG	180
TNFA	AGGCACTCCCCCAAAA-GATG	GCTCCTCCACTT-GGTGGTTT	219
BDNF	GGCGAGCAGAG-TCCATTCAG	TCGTCAGAC-CTCTCGAACCT	614
VEGFA-	AGGAGGAGGG-CAGAATCATCA	GGCACACAGGATGGCTTGAA	144
ITGA2	GGTCATCAGGGCAC-TATCCG	GGCTCCTAAAGGCTCCATCG	70
ITGA3	GGGC-TACCCTATTCCTCCGA	CAGCTCCGAG-TCAATGTCCA	285
ITGAV	TCCCATCAGTGGTTT-GGAGC	AGCTGAC-GTGATCTACATGG	154
HSP60	GGACGCTGACGCGAAGA	TAAGCCCGAGTGAGATGAGGA	153
KLF4	TGCGGCAAAAC-CTACACAAAG	GTTCATCTGAGCGGGCGAAT	121

TableS2. Primers used for analysis of miRNA expression

miRNA	Stem-loop primer	Forward primer	Reverse primer
U6 snRNA	CGCTTCACGAATTT-GCGTGTCA	GCTTCGG-CAGCACATA-TACTAAAAT	CGCTTCAC-GAATTT-GCGTGTCAT
miR-7	CAC-CGTTCCCCGCCGTCGGTGACAACA	CGCCCTG-GAAGACTAG-TGAT	CCGTCGGTGACAACAAAAT
miR-21	GTCGTATCCAGTG-CAGGGTCCGAGGTATTCGCACTGGATAC-GACTCAACA	GCCCGCTAGCTTATCAGACTGATG	CAGTG-CAGGGTCCGAGGT
miR-31	GTCGTATCCAGTG-CAGGGTCCGAGGTATTCGCACTGGATAC-GACAGCTAT	GCCG-CAGGCAA-GATGCTGGC	
miR-96	GTCGTATCCAGTG-CAGGGTCCGAGGTATTCGCACTGGATAC-GACAGCAAA	GCCCGCTTT-GGCACTAG-CACATT	

miR-135b	GTCGTATCCAGTG-CAGGGTCCGAGGTATTCGCACTGGATAC-GACTCACAT	GCCCGC-TATGGCTTTCATTCCT	
miR-203	GTCGTATCCAGTG-CAGGGTCCGAGGTATTCGCACTGGATAC-GACCTAGTGGTC	GTATCCAG-TG-CAGGGTCCGA	CGAC-GGTGAAATGTTTAG
miR-221	CAC-CGTTCCCCGCCGTCGGTGGAAACC	CGGGCAGC-TACATT-GTCTG	CGTCGGTG-GAAAC-CAGCA
miR-451	CAC-GGAACCCCGCCGACCGTGAACTCA	CGCCGAAAC-CGTTACCAT	GCCGAC-CGTGAACTCAGTAAT

Table S3. The siRNA sequences

**Gene**	**siRNA sequence**	
	**sense**	**antisense**
EGFR	UGAUCUGUCACCACAUAAUUACGG	CCCGUAAUUAUGUGGUGACAGAUCA
	UUAGAUAAGACUGCUAAGGCAUAGG	CCUAUGCCUUAGCAGUCUUAUCUAA
	UUUAAAUUCACCAAUACCUAUUCCG	CGGAAUAGGUAUUGGUGAAUUUAAA
Scramble	UUCUCCGAACGUGUCACGU	ACGUGACACGUUCGGAGAA

Table S4. Analysis of mRNA contained in EVs derived from the mel H cells cultivated at pH 6.5 and pH 7.4. Expression is shown as normalized mRNA level ± SEM (*n* = 8).

**Gene**	**EVs (pH 7.4)**	**EVs (pH 6.5)**
EGFR	-	-
PDGFRA	-	-
TNFA	-	-
BDNF	-	-
VEGFA	-	$1.14 \times 10^{-6} \pm 5.2 \times 10^{-7}$
ITGA2	-	-
ITGA3	-	$2.92 \times 10^{-5} \pm 4.3 \times 10^{-6}$
ITGV	-	$2.42 \times 10^{-6} \pm 1.3 \times 10^{-7}$
HSP60	-	$1.99 \times 10^{-8} \pm 1.8 \times 10^{-9}$

Table S5. Analysis of mRNA contained in EVs derived from the mel Kor cells cultivated at pH 6.5 and pH 7.4. Expression is shown as normalized mRNA level ± SEM (*n* = 8).

**Gene**	**EVs (pH 7.4)**	**EVs (pH 6.5)**
EGFR	-	-
PDGFRA	-	-

TNFA	-	-
BDNF	$2.56 \times 10^{-10} \pm 1.5 \times 10^{-10}$	-
VEGFA	$3 \times 10^{-7} \pm 4.9 \times 10^{-8}$	$8.21 \times 10^{-12} \pm 7 \times 10^{-12}$
ITGA2	-	$5.06 \times 10^{-6} \pm 4.5 \times 10^{-7}$
ITGA3	-	$1.52 \times 10^{-5} \pm 4.5 \times 10^{-6}$
ITGV	-	$3.6 \times 10^{-6} \pm 4.6 \times 10^{-7}$
HSP60	-	$4.34 \times 10^{-7} \pm 1.3 \times 10^{-7}$

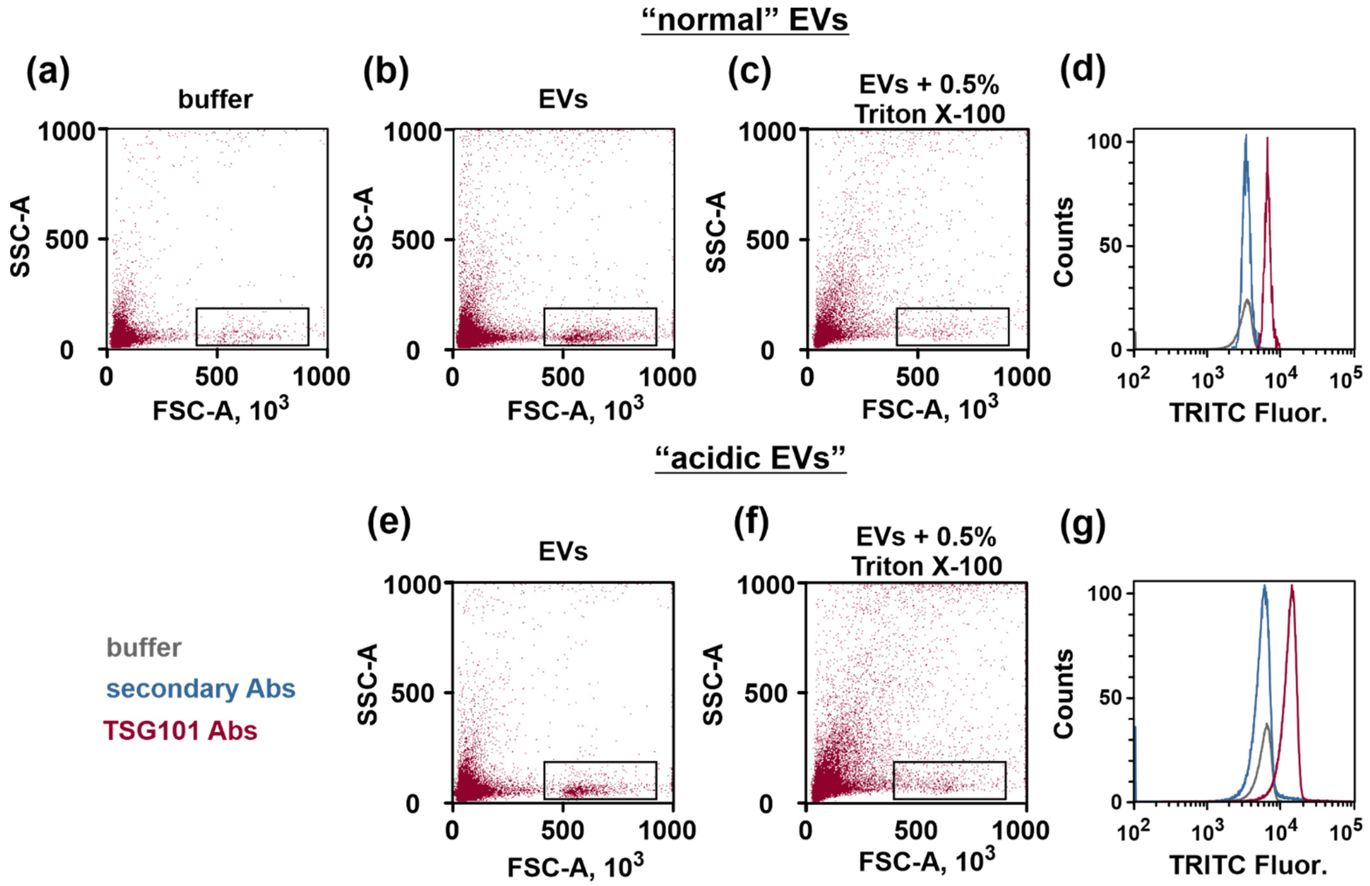

Figure S1. Exosome characterization by flow cytometry: the double-filtered FC buffer (**a**); "normal" (**b**) and "acidified" (**e**) EVs; "normal" (**c**) and "acidified" (**f**) EVs after the 30 min incubation with Triton X-100; "normal" (**d**) and "acidified" (**g**) EVs stained with the antibody to TGS101 are shown.

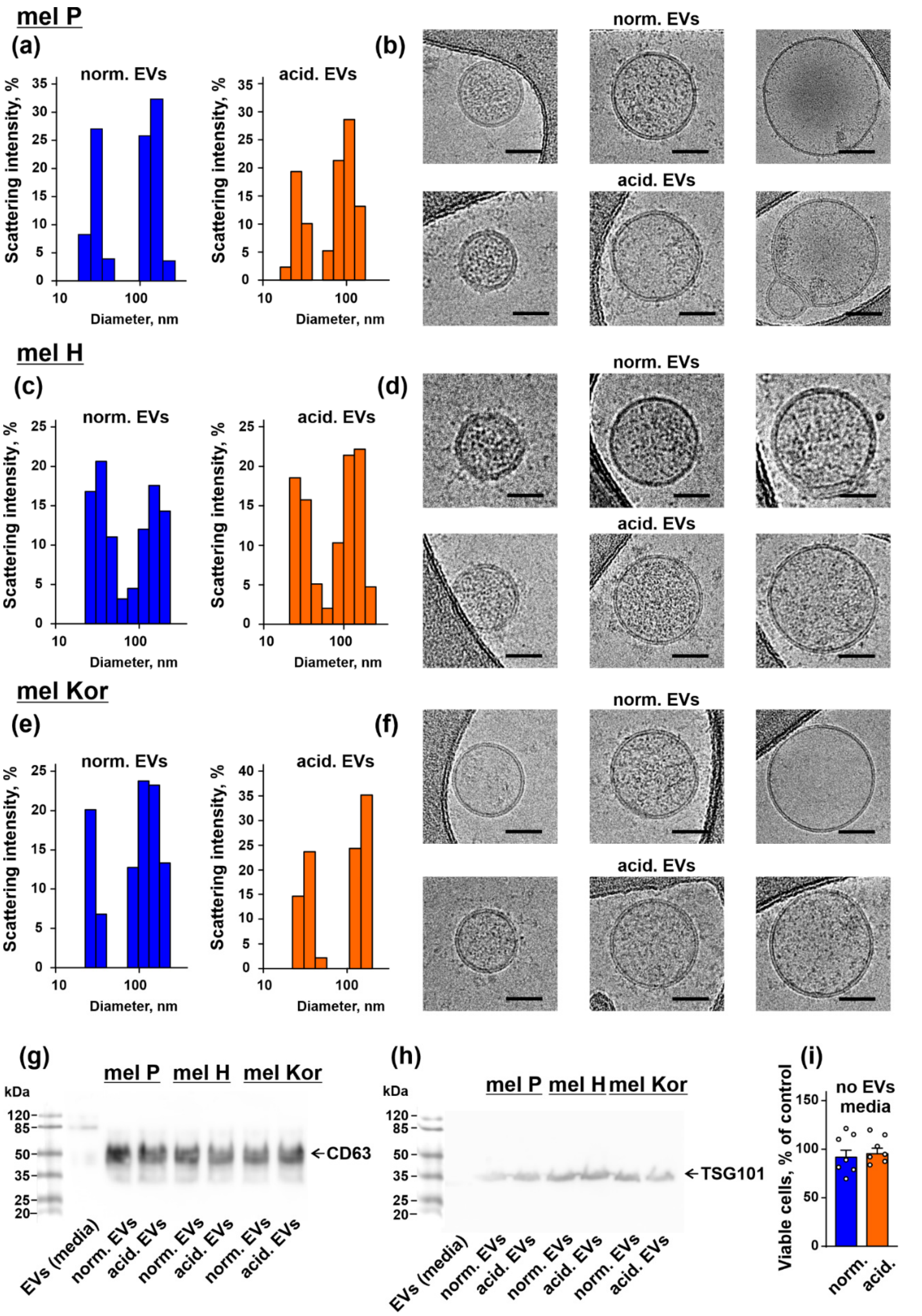

Figure S2. Characterization of EVs derived from the mel P (**a, b**), mel H (**c, d**), and mel Kor (**e, f**) cells: EVs size analysis according to dynamic light scattering (**a,c,e** for mel P, mel H and mel Kor cells, respectively), EVs visualization by cryo-EM (**b, d, e** for mel P, mel H and mel Kor cells, respectively, scale bar 25 nm); **g, h** – analysis of the expression of the exosome markers CD63 (**g**) and TSG101 (**h**) in EVs by Western blotting. EVs from the media (without cells) was used as the negative control; (**i**) influence of "EVs" from the FCS-supplemented media on growth of the Het-1A cells. Data presented as % of the control (untreated cells) ± SEM (n = 7).

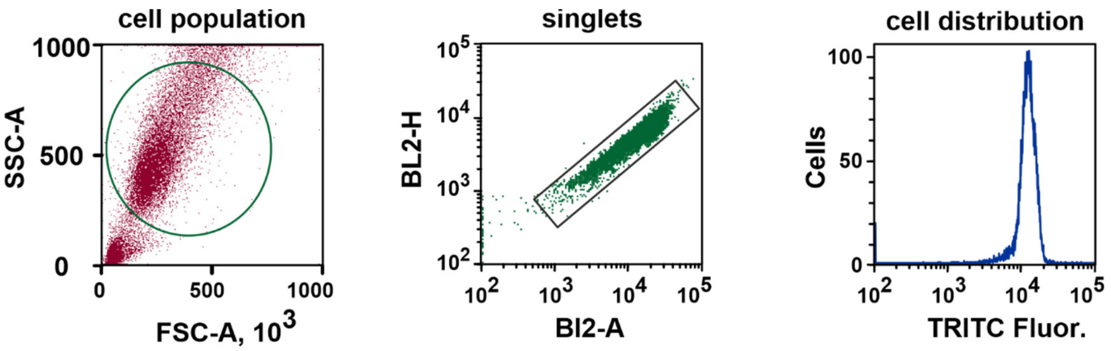

Figure S3. Gating strategy for the analysis of the EVs influence on the expression of the prooncogenic markers in the keratinocytes.

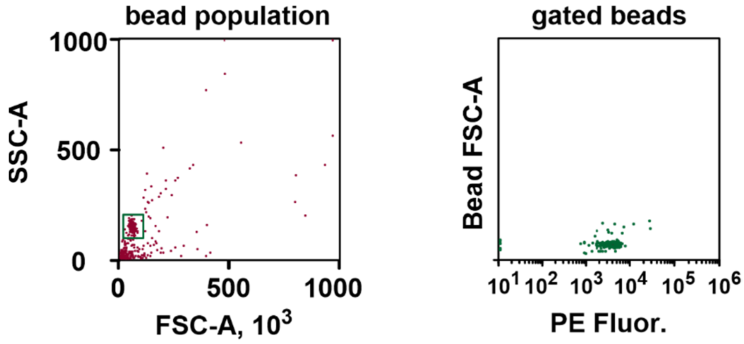

Figure S4. Gating strategy for the analysis by the Bio-Plex magnetic bead assay of the intracellular pathways activated in the keratinocytes.

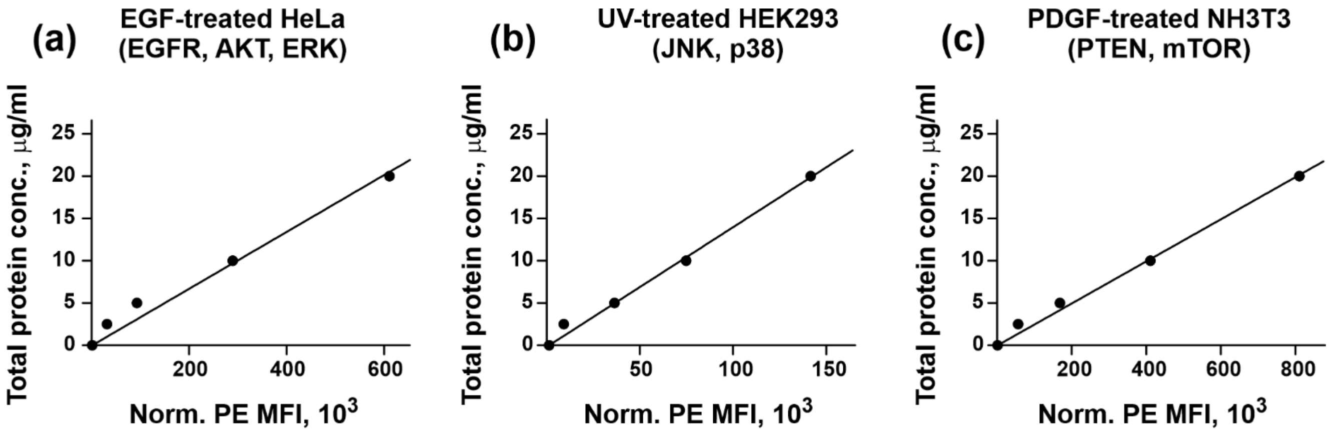

Figure S5. The Bio-Plex assay linearity analysis. Cell lysates were serially diluted and the protein phosphorylation was detected according to the manufacturer protocol. After that the linear regression was performed in the GraphPad Prism 9.2 software.

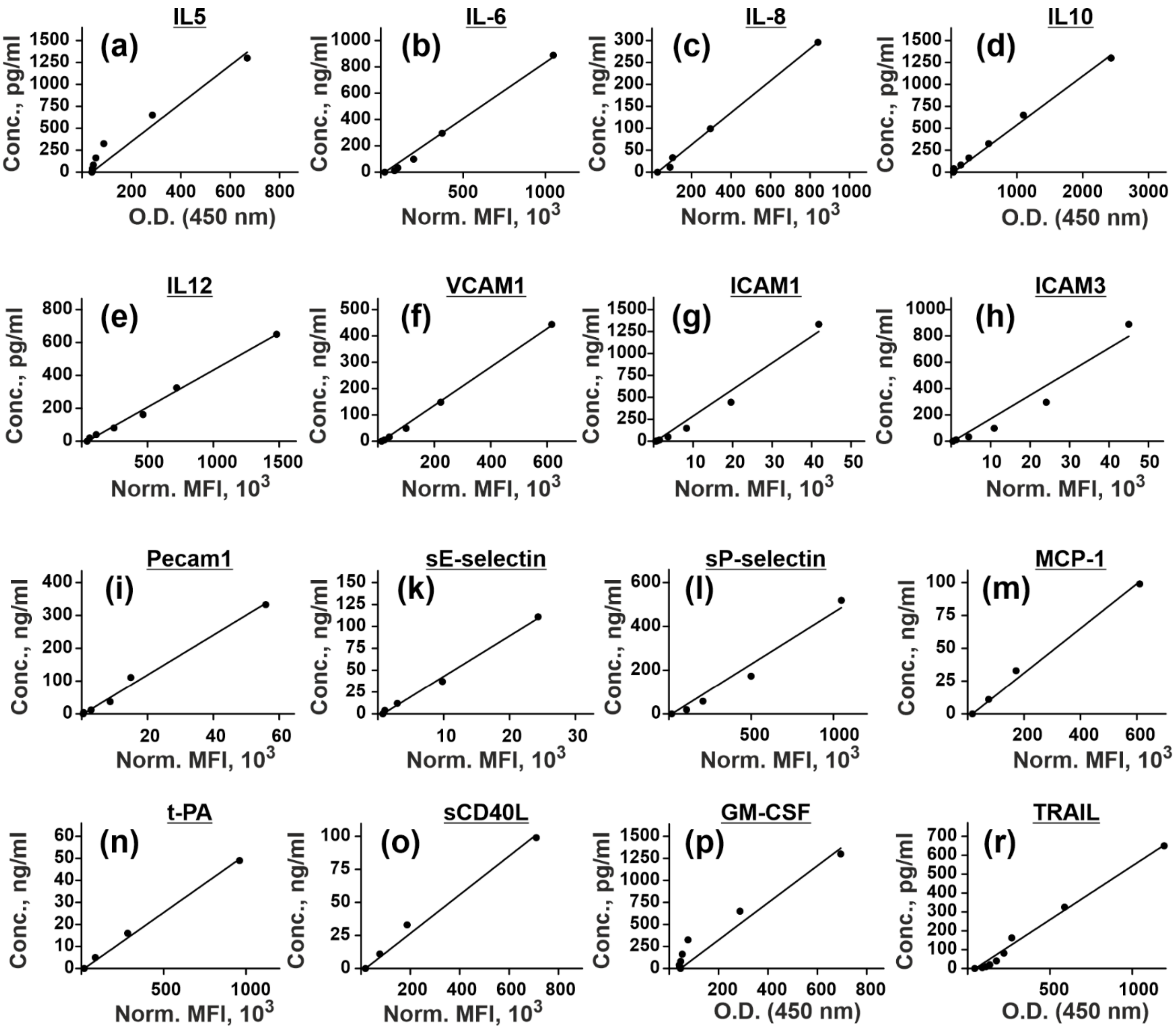

Figure S6. The ELISA's and Flow Cytomix kits assays linearity analysis. Serial dilution of the standard proteins was subjected to the ELISA or Flow cytomix assays according to the manufacturer instructions. Concentration of IL5 (**a**), IL10 (**d**), IL12 (**e**), GM-CSF (**p**), and TRAIL (**r**) was assayed by ELISA. Concentration of IL6 (**b**), IL8 (**c**), sVCAM-1 (**f**), sICAM-1 (**g**), sICAM-3 (**h**), sPECAM-1 (**i**), sE-selectin (**k**), sP-selectin (**l**), MCP-1 (**m**), t-PA (**n**), and sCD40L (**o**) was analyzed by the Flow Cyto-mix kits. After that the linear regression was performed in the GraphPad Prism 9.2 software.

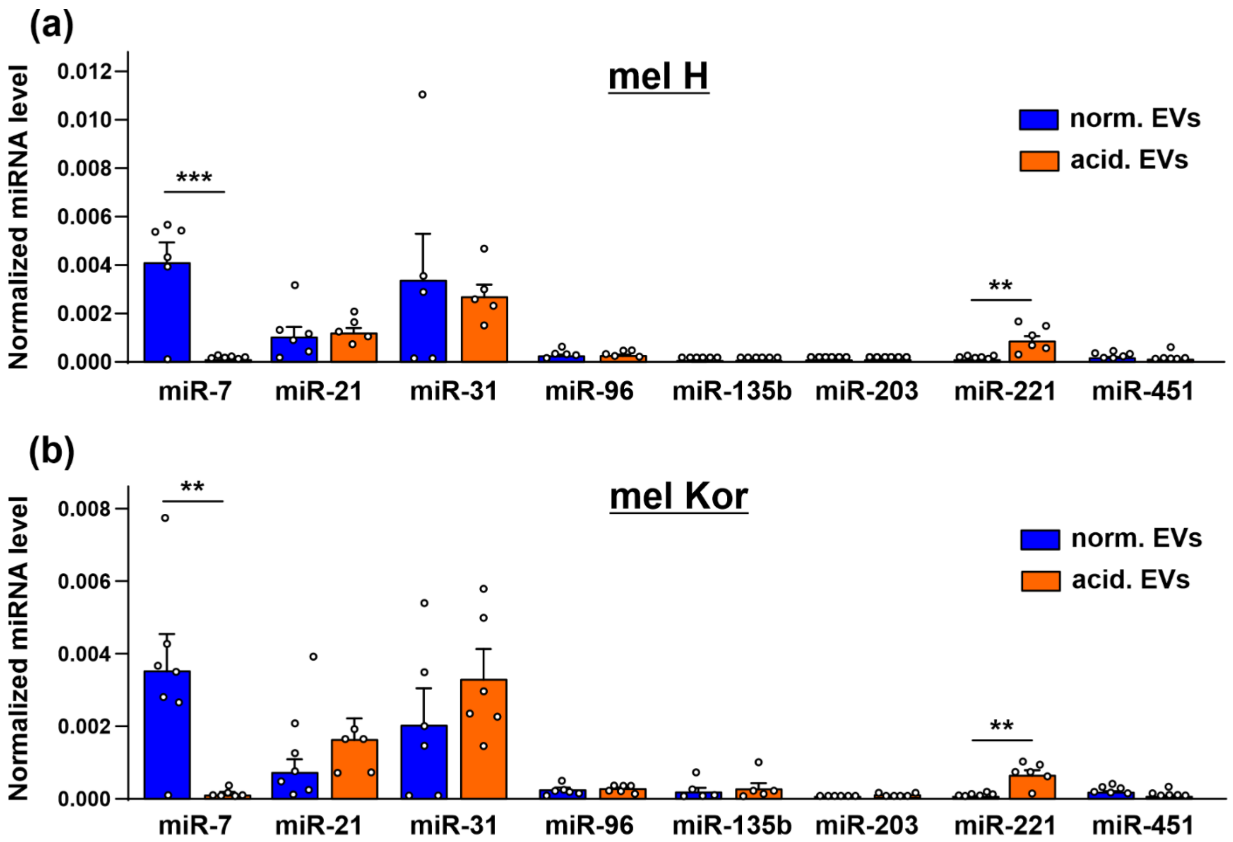

Figure S7. Analysis of the miRNA expression in EVs derived from the metastatic melanoma cells mel H (**a**) and mel Kor (**b**) cultivated at pH 6.5 ("acidified") and pH 7.4 ("normal"): Expression of different miRNA in "normal" and "acidified" EVs was assayed by qPCR with stem-loop primers and normalized to the U6 non-coding RNA. Data presented as the relative miRNA level ± SEM (n = 5-6). ** ($p < 0.01$) and *** ($p < 0.001$) indicate significant difference between the data groups according to the two-tailed t-test.

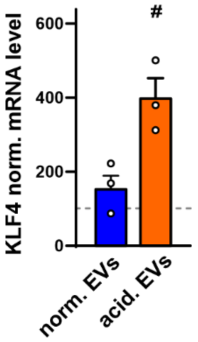

Figure S8. PCR analysis of the *KLF* expression in the keratinocytes treated by "normal" and "acidified" EVs derived from the mel P cells. *KLF4* expression was normalized to the expression of the *RPL13a* and *S18* genes and presented as the relative expression ± SEM (n = 3). # ($p < 0.05$) indicates significant difference between the treated and untreated cells (control, shown by dashed line) according to the one sample t-test.

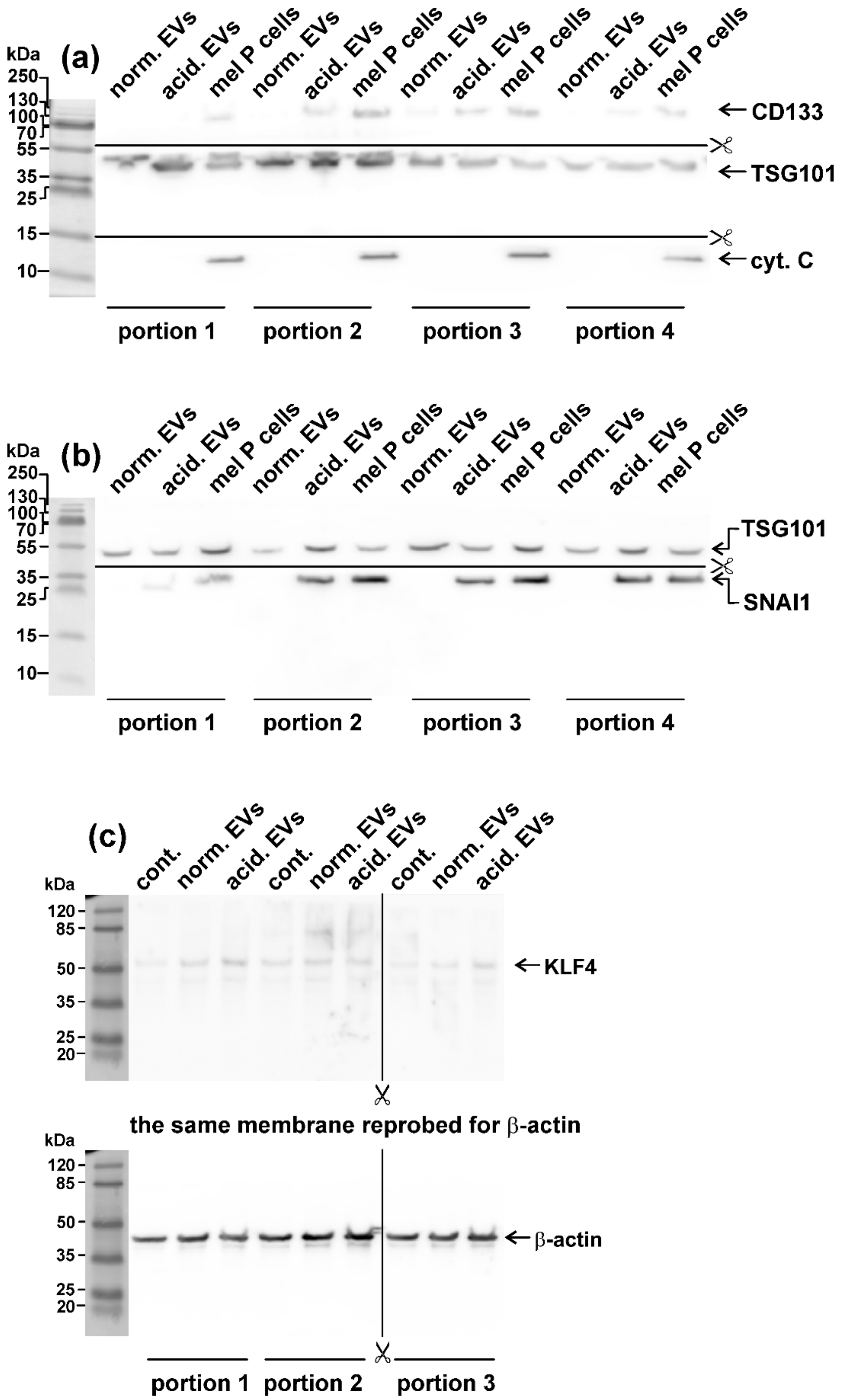

Figure S9. Whole membranes used for the analysis of the CD133 (**a**), SNAI1 (**b**), and KLF (**c**) expression by Western blotting. TSG101 was used as the exosomal marker, cytochrome C (**a**) served as the negative and positive control for the protein detection in EVs and the mel P cells, respectively.

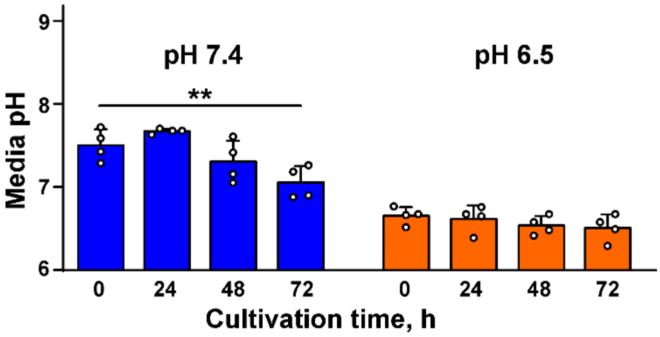

Figure S10. Media pH changes upon the cultivation of the mel P cells in the "normal" and "acidic" media (n = 4). ** (p < 0.01) indicates significant difference between the data groups by the one-way ANOVA followed by Tukey's post hoc test.

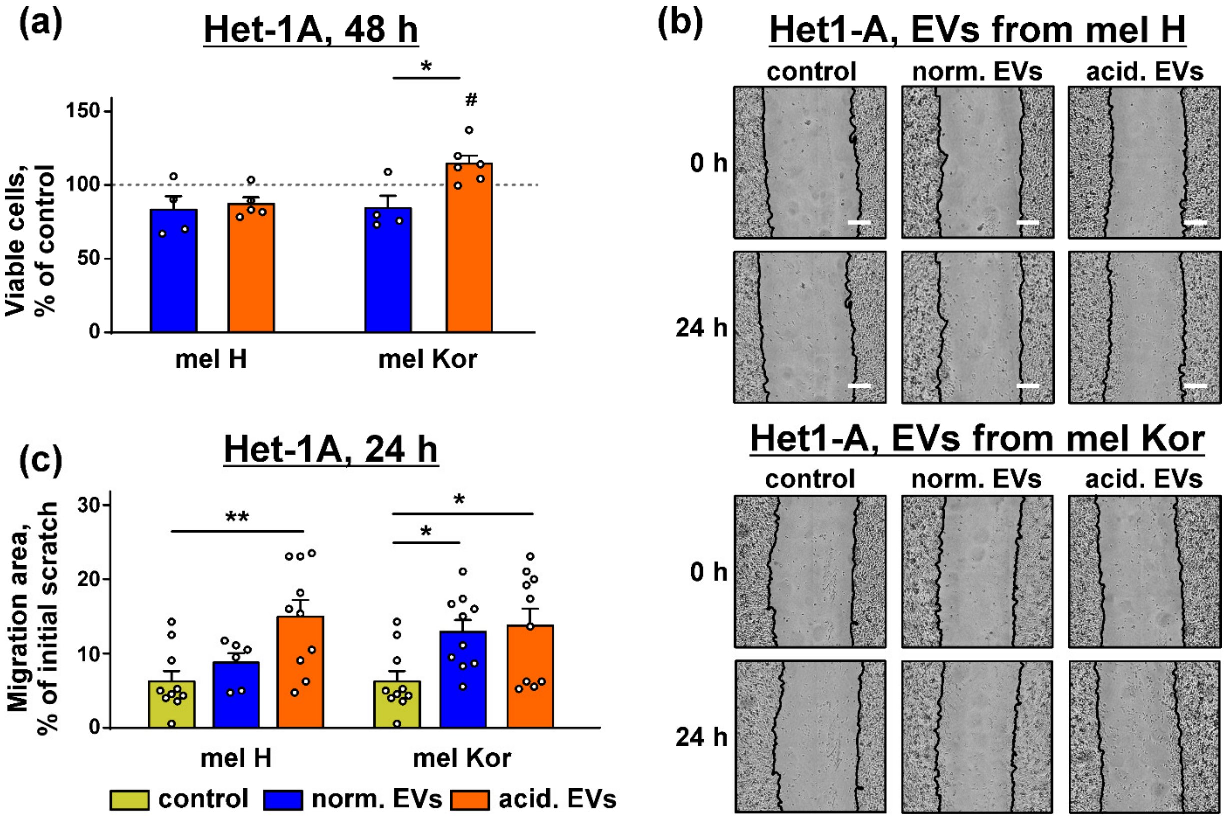

Figure S11. Effect of "normal" and "acidified" EVs on growth and migration of the keratinocytes. (**a**) Influence of "normal" and "acidified" EVs on viability of the Het-1A cells upon the 48 h incubation according to the WST-1 assay. Data were normalized to viability of the untreated cells (control, shown by dashed line). Data are % of the untreated cells ± SEM (n = 4-6), # (p < 0.05) indicates significant difference from the untreated cells by the one-sample t-test, * (p < 0.05) indicates significant difference between the data groups by the two-tailed t-test; (**b**)representative pictures of the scratch test for the Het-1A cells upon the 24 h incubation with "normal" and "acidified" EVs derived from the mel H and mel Kor cells; (**c**) scratch square occupied by the migrating Het-1A cells. Data are presented as % of the scratch surface, occupied by the migrating cells ± SEM (n = 6-10), * (p < 0.05) and ** (p < 0.01) indicate significant difference between the data groups by the one-way ANOVA followed by Tukey's post hoc test.

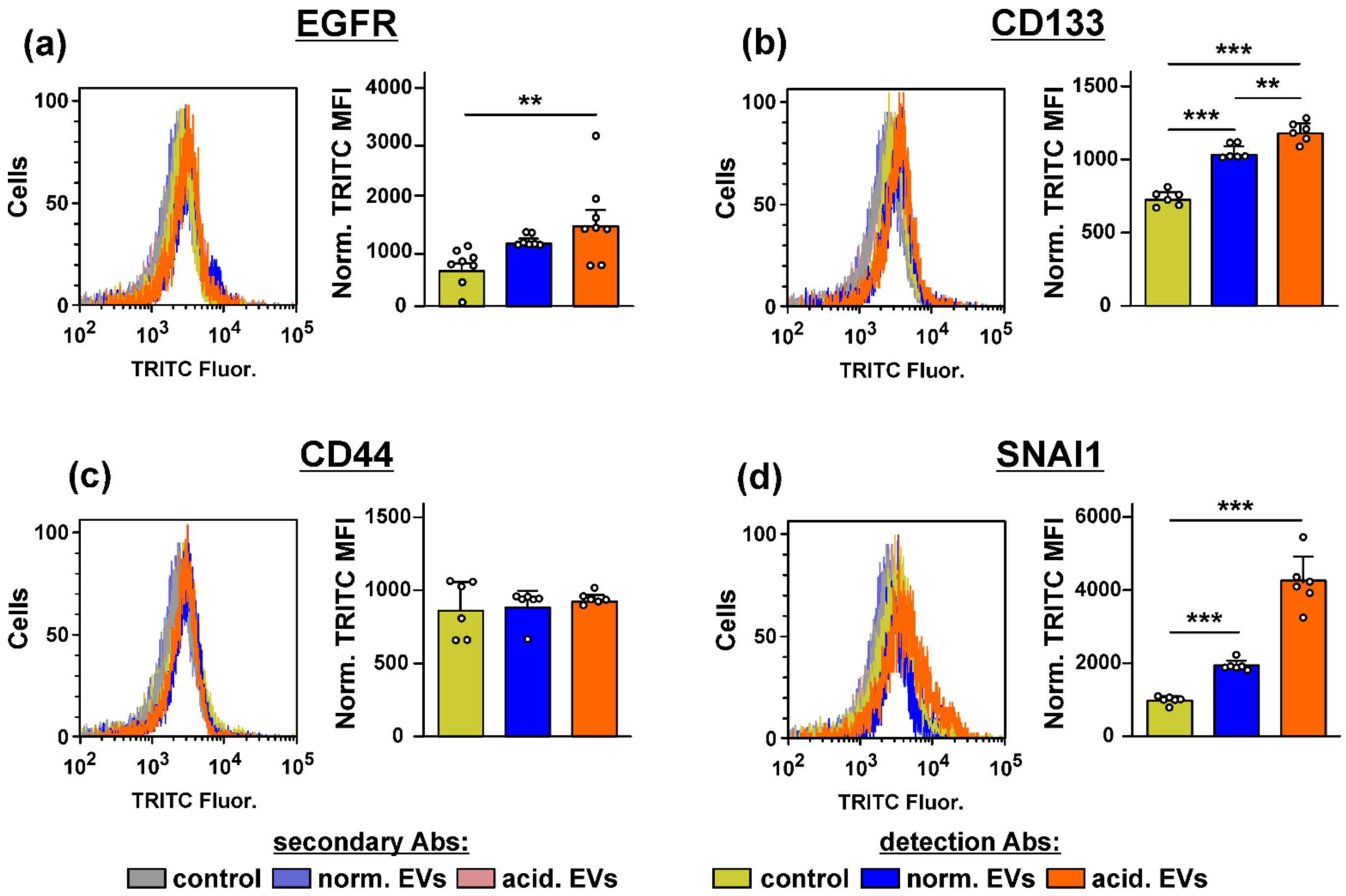

Figure S12. Influence of "normal" and "acidified" EVs on the expression of the EGFR (**a**), CD133 (**b**), CD44 (**c**), and SNAI1 (**d**) proteins in the keratinocytes. Representative cell distribution histograms of the EGFR, CD133, CD44, and SNAI1 proteins stained in the keratinocytes and the expression level of the corresponding proteins are shown on the left and right panels, respectively. Data presented as normalized MFI ± SEM (n = 6). ** ($p < 0.01$) and *** ($p < 0.001$) indicate significant difference between the data groups by the one-way ANOVA followed by Tukey's post hoc test.

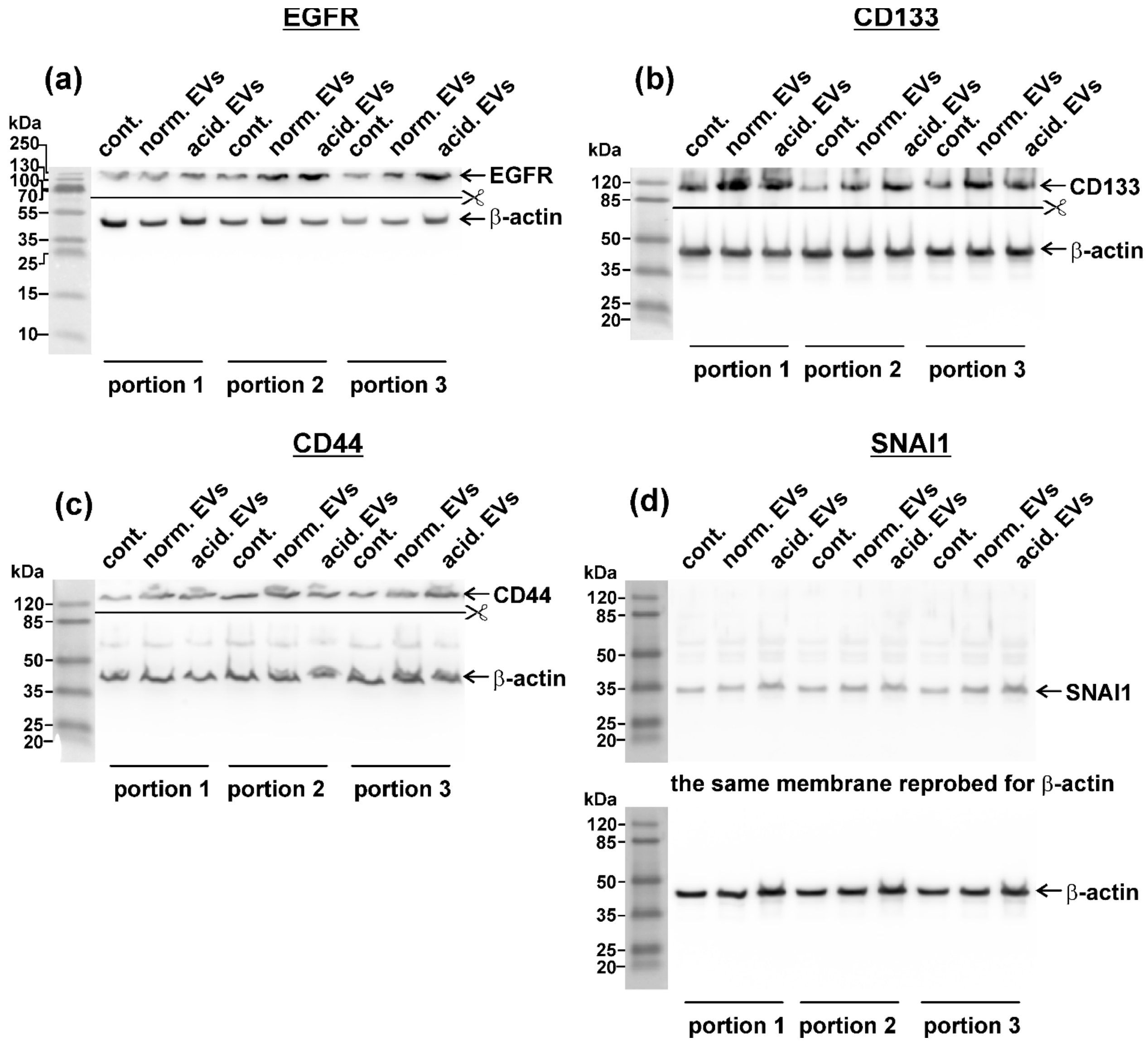

Figure S13. Whole membranes used for the analysis of the influence of "normal" and "acidified" EVs on the expression of the EGFR (**a**), CD133 (**b**), CD44 (**c**), and SNAI1 (**d**) proteins in the keratinocytes.
